# Supplementary figures and images for: Incorporating connectivity among Internet search data for enhanced influenza-like illness tracking
Source: PLoS One. 2024 Aug 26;19(8):e0305579. doi: 10.1371/journal.pone.0305579 (PMC11346739; doi:10.1371/journal.pone.0305579)

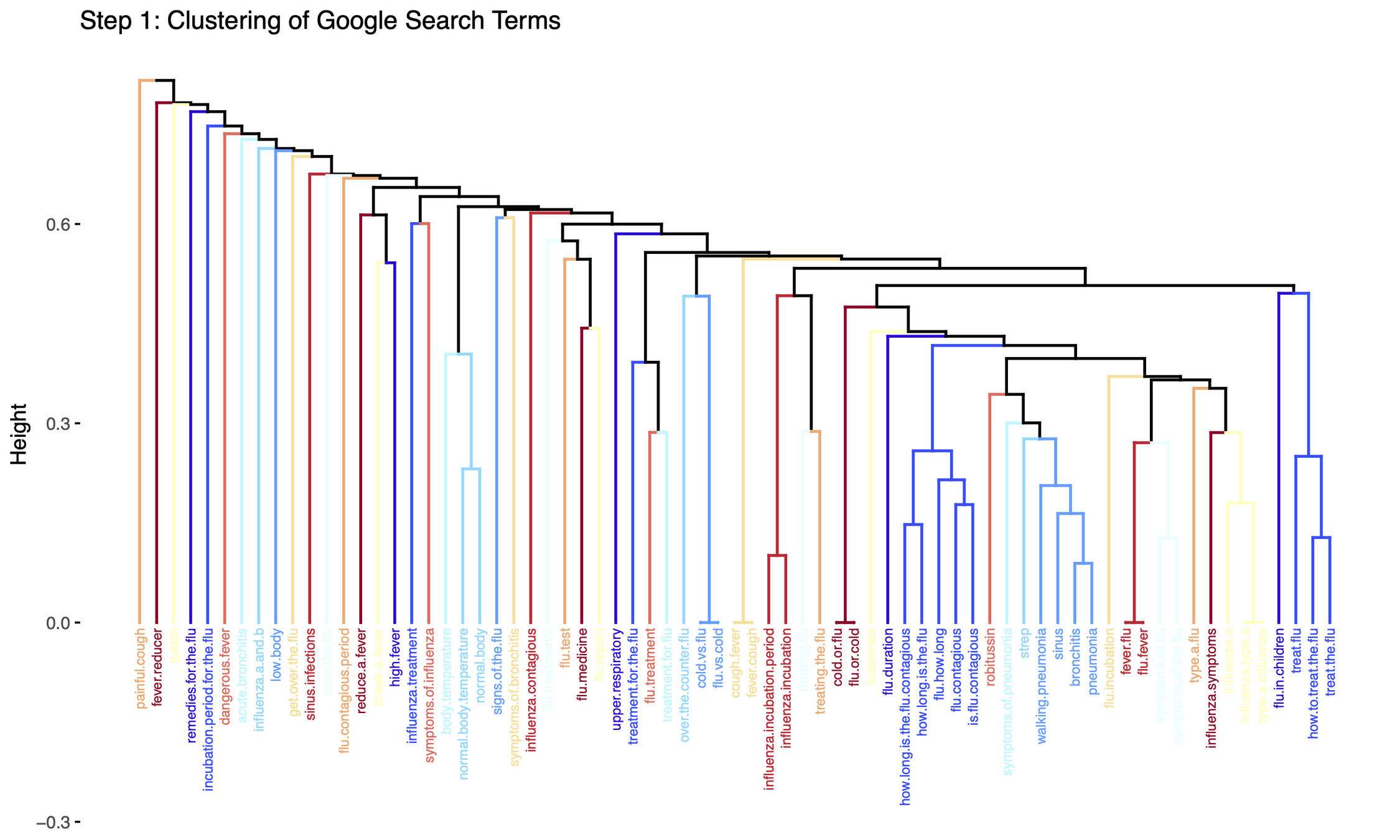

Supplement: S1 Fig — 53 clusters were identified. Hierarchical clustering with average linkage and correlation distance metric were used. The clustering was conducted based on the time series of Google search data from January 10, 2004 (the earliest available Google Trends data) to March 29, 2009 (the earliest prediction date by these 71 terms). (TIF) [file pone.0305579.s001.tif]

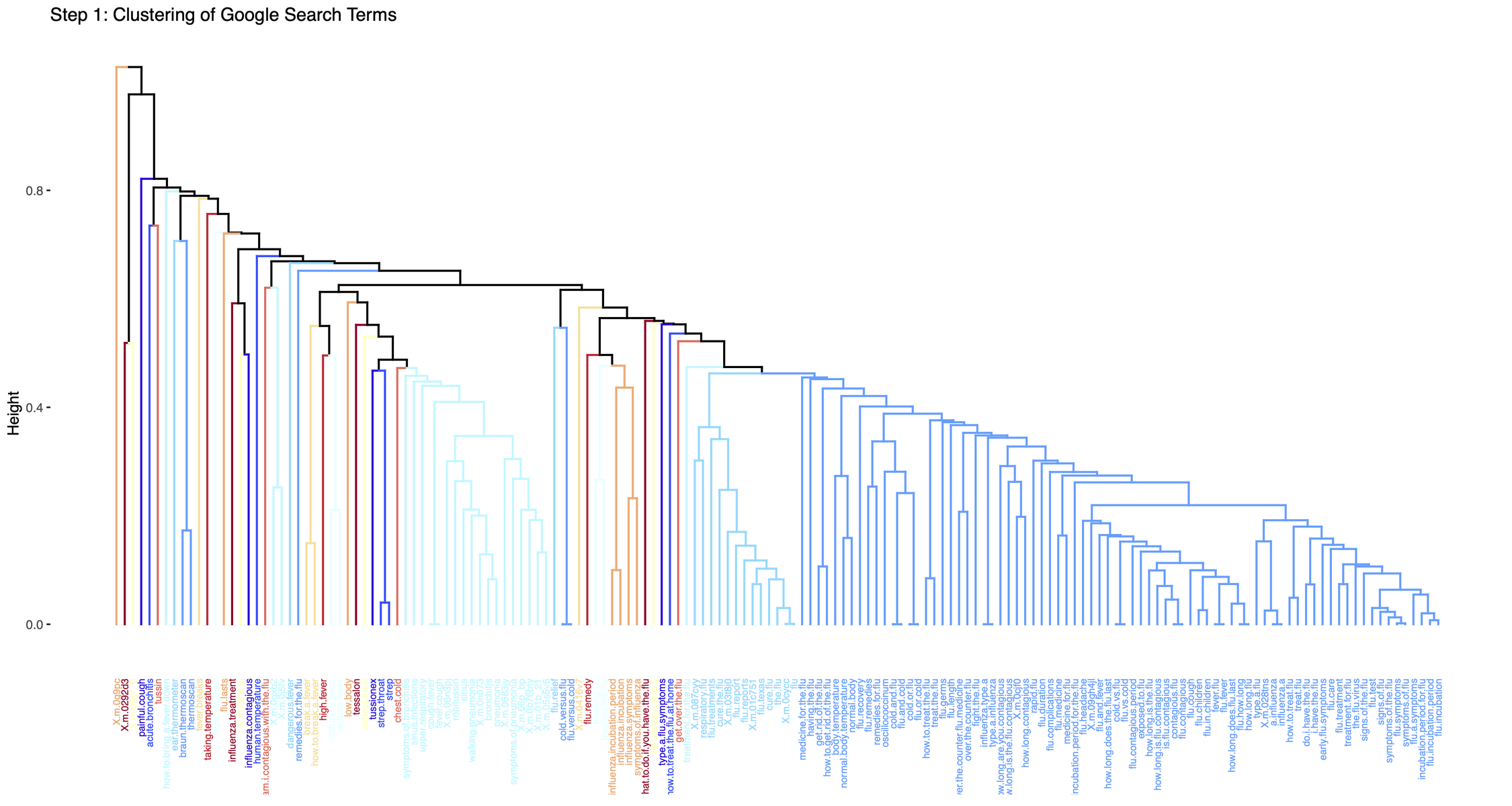

Supplement: S2 Fig — 45 clusters were identified. Hierarchical clustering with average linkage and correlation distance metric were adopted. The clustering was conducted based on the time series of Google search data from January 10, 2004 (the earliest available Google Trends data) to May 22, 2010 (the earliest prediction date by these 161 terms). (TIF) [file pone.0305579.s002.tif]

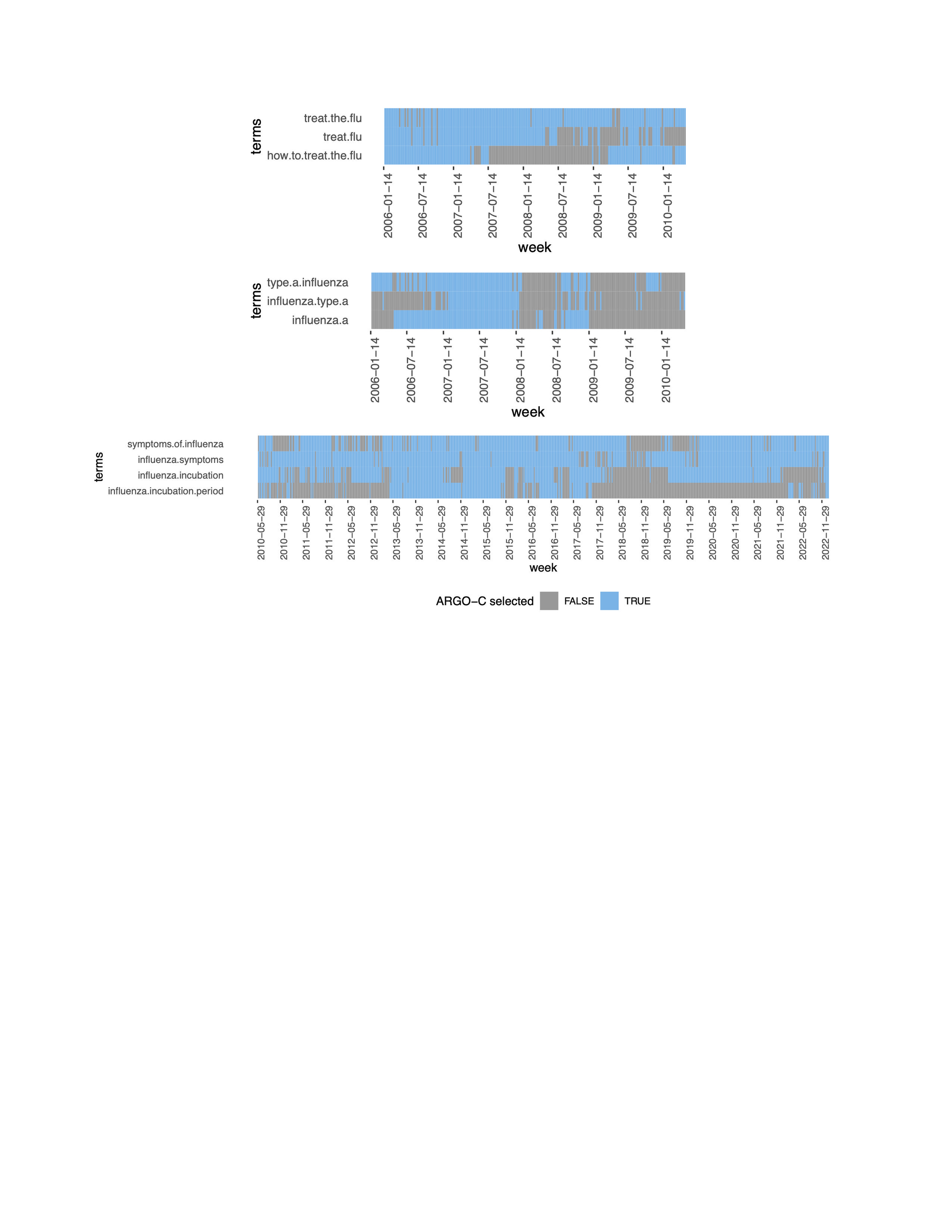

Supplement: S3 Fig — The heatmaps indicate whether each predictor was included in the predictive ARGO-C model at each week. Three exemplary clusters were highlighted. Each of the top two clusters contains three search terms (identified among 71 search terms by March 29, 2009, used for predictions before 2010), while the last one includes four search terms (identified among 161 topics/terms by May 22, 2010 and used for predictions since 2010). The entire cluster was penalized and excluded from the model when an entire column in the traceplot is colored grey. (TIF) [file pone.0305579.s003.tif]
