# Supplementary material for: Incorporating connectivity among Internet search data for enhanced influenza-like illness tracking
Source: PLoS One. 2024 Aug 26;19(8):e0305579. doi: 10.1371/journal.pone.0305579 (PMC11346739; doi:10.1371/journal.pone.0305579)
Supplement: S1 Table — Clustering is based on the 161 Google search terms collected by May 22, 2010, with 45 clusters. Methods in comparison include hierarchical clustering (HC) with average linkage (ave), with complete linkage (comp), and single linkage (single), based on correlation/Pearson distance metric, as well as Kmeans and PAM. Metrics for comparison include within-cluster sum of squares (WSS), silhouette, and gap statistics. Note that for correlation-based distance, WSS and gap statistics (defined based on Euclidean distance) are less relevant. All clusterng methods can be readily applied for ARGO-C. Based on the superior performance in this evaluation, we use hierarchical clustering with average linkage as default in this paper. (PDF) [file pone.0305579.s004.pdf]

	HC (ave)	Kmeans	PAM	HC (comp)	HC (single)
Silhouette	**0.115**	0.085	0.087	0.113	0.025
WSS	**17371.886**	18601.143	20024.194	19536.180	27494.266
Gap	0.648	**0.662**	0.648	0.631	0.570

Table S1. Evaluation of various clustering methods for grouping search terms. Clustering is based on the 161 Google search terms collected by May 22, 2010, with 45 clusters. Methods in comparison include hierarchical clustering (HC) with average linkage (ave), with complete linkage (comp), and single linkage (single), based on correlation/Pearson distance metric, as well as Kmeans and PAM. Metrics for comparison include within-cluster sum of squares (WSS), silhouette, and gap statistics. Note that for correlation-based distance, WSS and gap statistics (defined based on Euclidean distance) are less relevant. All clusterng methods can be readily applied for ARGO-C. Based on the superior performance in this evaluation, we use hierarchical clustering with average linkage as default in this paper.
