# Supplementary material for: Incorporating connectivity among Internet search data for enhanced influenza-like illness tracking
Source: PLoS One. 2024 Aug 26;19(8):e0305579. doi: 10.1371/journal.pone.0305579 (PMC11346739; doi:10.1371/journal.pone.0305579)
Supplement: S2 Table — The evaluation is based at the national level %ILI in multiple periods and multiple metrics. RMSE, MAE, and correlation are reported. The method with the best performance is highlighted in boldface for each metric in each period. Methods considered here include ARGO-C, VAR1, GFT, the original ARGO, and the naive method. All comparisons are conducted on the original scale of the CDC’s %ILI. The overall period ’09-’23 is March 29, 2009 to January 28, 2023, including the period since COVID. Each regular flu season is from week 40 to week 20 next year, as defined by CDC’s Morbidity and Mortality Weekly Report. (The ’22-’23 season is up to January 28, 2023). (PDF) [file pone.0305579.s005.pdf]

	Overall '09-'23	'20-'21	'21-'22	'22-'23
RSME				
ARGO-C	**0.252**	0.113	**0.224**	0.490
GFT	–	–	–	–
VAR1	0.359	**0.081**	0.357	0.769
ARGO	0.258	0.100	0.232	**0.487**
naive	0.355	0.082	0.358	0.734
MAE				
ARGO-C	0.161	0.089	0.181	0.429
GFT	–	–	–	–
VAR1	0.211	**0.066**	0.245	0.644
ARGO	**0.158**	0.086	**0.168**	**0.374**
naive	0.210	0.067	0.239	0.591
Correlation				
ARGO-C	**0.984**	0.927	**0.983**	**0.975**
GFT	–	–	–	–
VAR1	0.968	**0.941**	0.899	0.909
ARGO	0.983	0.940	0.962	0.969
naive	0.968	0.939	0.900	0.907

Table S2. Comparison of % ILI estimation between ARGO-C and other benchmarks at the national level, for flu seasons since COVID-19. The evaluation is based at the national level %ILI in multiple periods and multiple metrics. RMSE, MAE, and correlation are reported. The method with the best performance is highlighted in boldface for each metric in each period. Methods considered here include ARGO-C, VAR1, GFT, the original ARGO, and the naive method. All comparisons are conducted on the original scale of the CDC's %ILI. The overall period '09-'23 is March 29, 2009 to January 28, 2023, including the period since COVID. Each regular flu season is from week 40 to week 20 next year, as defined by CDC's Morbidity and Mortality Weekly Report. (The '22-'23 season is up to January 28, 2023).
