# Supplementary material for: Incorporating connectivity among Internet search data for enhanced influenza-like illness tracking
Source: PLoS One. 2024 Aug 26;19(8):e0305579. doi: 10.1371/journal.pone.0305579 (PMC11346739; doi:10.1371/journal.pone.0305579)
Supplement: S3 Table — The evaluation period is March 29, 2009 to February 29, 2020, before COVID. RMSE is reported for varying α, the weight between Lasso penalty and group Lasso penalty in the ARGO-C model, with α = 1 corresponding to vanilla ARGO without group penalty. (PDF) [file pone.0305579.s006.pdf]

α	0.1	0.3	0.5	0.7	0.9	0.95	1 (ARGO)	naive
RMSE	0.216	0.217	0.219	0.217	0.220	0.220	0.238	0.352

Table S3. Comparison of % ILI estimation by ARGO-C and other benchmarks at the national level, with varying tuning parameter α. The evaluation period is March 29, 2009 to February 29, 2020, before COVID. RMSE is reported for varying α, the weight between Lasso penalty and group Lasso penalty in the ARGO-C model, with $\alpha = 1$ corresponding to vanilla ARGO without group penalty.
