# Supplementary material for: Incorporating connectivity among Internet search data for enhanced influenza-like illness tracking
Source: PLoS One. 2024 Aug 26;19(8):e0305579. doi: 10.1371/journal.pone.0305579 (PMC11346739; doi:10.1371/journal.pone.0305579)
Supplement: S4 Table — The evaluation period is March 29, 2009 to February 29, 2020, before COVID. The relative accuracy, characterized by the ratio predication accuracy of ARGO-C over ARGO is reported in relative RMSE, MAE, and Correlation. Bootstrap is conducted to estimate the relative accuracy, with the bootstrapped SE and quantiles reported (based on 50 bootstrapped samples). For RMSE and MAE, a relative accuracy <1 indicates advantage; for correlation, a relative accuracy >1 indicates advantage. (PDF) [file pone.0305579.s007.pdf]

	Relative Accuracy (SE)	10%	25%	50%	75%	90%
RMSE	0.9291 (0.0504)	0.8737	0.8914	0.9273	0.9580	0.9851
MAE	0.9720 (0.0364)	0.9271	0.9473	0.9731	0.9985	1.0133
Correlation	1.0028 (0.0015)	1.0006	1.0021	1.0029	1.0037	1.0045

Table S4. Relative accuracy between ARGO-C and ARGO on the national level.The evaluation period is March 29, 2009 to February 29, 2020, before COVID. The relative accuracy, characterized by the ratio predication accuracy of ARGO-C over ARGO is reported in relative RMSE, MAE, and Correlation. Bootstrap is conducted to estimate the relative accuracy, with the bootstrapped SE and quantiles reported (based on 50 bootstrapped samples). For RMSE and MAE, a relative accuracy < 1 indicates advantage; for correlation, a relative accuracy > 1 indicates advantage.
