# Supplementary material for: Incorporating connectivity among Internet search data for enhanced influenza-like illness tracking
Source: PLoS One. 2024 Aug 26;19(8):e0305579. doi: 10.1371/journal.pone.0305579 (PMC11346739; doi:10.1371/journal.pone.0305579)
Supplement: S5 Table — The evaluation period is March 29, 2009 to February 29, 2020, before COVID. RMSE is reported. ARGO-C (random) is based on randomly assigned clusters in the Step 1 of ARGO-C with the same number of clusters as identified by unsupervised learning in ARGO-C; the RMSE is an averaged of 10 random assignments. ARGO (single) is based on one single cluster including all search terms. ARGO (group) is based on group-aggregated search frequencies, where each predictor is simply the sum of frequencies of search terms in each cluster. (PDF) [file pone.0305579.s008.pdf]

	ARGO-C	ARGO-C (random)	ARGO-C (single)	ARGO	ARGO (group)	naive
RMSE	0.246	0.254	0.260	0.261	0.287	0.352

Table S5. Comparison of % ILI estimation by ARGO-C and additional benchmarks at the national level. The evaluation period is March 29, 2009 to February 29, 2020, before COVID. RMSE is reported. ARGO-C (random) is based on randomly assigned clusters in the Step 1 of ARGO-C with the same number of clusters as identified by unsupervised learning in ARGO-C; the RMSE is an averaged of 10 random assignments. ARGO-C (single) is based on one single cluster including all search terms. ARGO (group) is based on group-aggregated search frequencies, where each predictor is simply the sum of frequencies of search terms in each cluster.
