# Supplementary material for: Incorporating connectivity among Internet search data for enhanced influenza-like illness tracking
Source: PLoS One. 2024 Aug 26;19(8):e0305579. doi: 10.1371/journal.pone.0305579 (PMC11346739; doi:10.1371/journal.pone.0305579)
Supplement: S6 Table — The evaluation is based on the average of 51 US state/district in multiple periods and multiple metrics. RMSE, MAE, and correlation are reported. The method with the best performance is highlighted in boldface for each metric in each period. Methods considered here include ARGO-C, VAR1, GFT, the original ARGOX, and the naive method. All comparisons are conducted on the original scale of the CDC’s %ILI. The overall period ’14-’23 is January 10, 2014 (first available estimate by ARGO framework) to January 28, 2023, including the period since COVID. The post-COVID period is the period since COVID, March 21, 2020 to January 28, 2023. Each regular flu season is from week 40 to week 20 next year, as defined by CDC’s Morbidity and Mortality Weekly Report. (The ’22-’23 season is up to January 28, 2023). (PDF) [file pone.0305579.s009.pdf]

	Overall '14-'23	post-COVID	'20-'21	'21-'22	'22-'23
RMSE					
ARGO-C	**0.564**	**0.554**	0.287	**0.480**	**1.048**
ARGOX	0.576	0.574	0.283	0.488	1.101
VAR1	1.789	1.660	0.736	1.368	3.398
GFT	–	–	–	–	–
naive	0.662	0.626	**0.279**	0.551	1.224
MAE					
ARGO-C	**0.325**	**0.304**	0.186	0.306	**0.685**
ARGOX	0.329	0.309	0.182	**0.305**	0.735
VAR1	1.114	0.979	0.511	0.954	2.364
GFT	–	–	–	–	–
naive	0.370	0.336	**0.166**	0.346	0.849
Correlation					
ARGO-C	**0.947**	**0.933**	0.703	**0.862**	**0.890**
ARGOX	0.944	0.929	0.709	0.855	0.877
VAR1	0.675	0.719	0.478	0.563	0.591
GFT	–	–	–	–	–
naive	0.929	0.919	**0.710**	0.816	0.844

Table S6. Comparison of % ILI estimation between ARGO-C and other benchmarks at the state level, for flu seasons since COVID-19. The evaluation is based on the average of 51 US state/district in multiple periods and multiple metrics. RMSE, MAE, and correlation are reported. The method with the best performance is highlighted in boldface for each metric in each period. Methods considered here include ARGO-C, VAR1, GFT, the original ARGOX, and the naive method. All comparisons are conducted on the original scale of the CDC's %ILI. The overall period '14-'23 is January 10, 2014 (first available estimate by ARGO framework) to January 28, 2023, including the period since COVID. The post-COVID period is the period since COVID, March 21, 2020 to January 28, 2023. Each regular flu season is from week 40 to week 20 next year, as defined by CDC's Morbidity and Mortality Weekly Report. (The '22-'23 season is up to January 28, 2023).
