# Supplementary material for: Incorporating connectivity among Internet search data for enhanced influenza-like illness tracking
Source: PLoS One. 2024 Aug 26;19(8):e0305579. doi: 10.1371/journal.pone.0305579 (PMC11346739; doi:10.1371/journal.pone.0305579)
Supplement: S7 Table — The coverage is for 95% nominal level. The average coverage over 51 states/city/district is 92.6%. The evaluation period is January 10, 2014 to March 21, 2020, excluding the period with COVID-19 influence. (PDF) [file pone.0305579.s010.pdf]

AL	AK	AZ	AR	CA	CO	CT	DE	DC	GA	HI	ID	IL	IN	IA
0.931	0.926	0.915	0.929	0.947	0.903	0.906	0.947	0.908	0.933	0.942	0.933	0.931	0.924	0.931
KS	KY	LA	ME	MD	MA	MI	MN	MS	MO	MT	NE	NV	NH	NJ
0.938	0.894	0.922	0.917	0.933	0.919	0.940	0.942	0.938	0.935	0.935	0.910	0.903	0.908	0.929
NM	NY	NC	ND	OH	OK	OR	PA	RI	SC	SD	TN	TX	UT	VT
0.915	0.899	0.952	0.949	0.924	0.924	0.926	0.940	0.919	0.938	0.940	0.915	0.933	0.931	0.945
VA	WA	WV	WI	WY	NYC									
0.954	0.908	0.917	0.945	0.885	0.915									

Table S7. Actual coverage of prediction intervals by ARGO-C for state-level %ILI prediction. The coverage is for 95% nominal level. The average coverage over 51 states/city/district is 92.6%. The evaluation period is January 10, 2014 to March 21, 2020, excluding the period with COVID-19 influence.
