# Supplementary material for: Incorporating connectivity among Internet search data for enhanced influenza-like illness tracking
Source: PLoS One. 2024 Aug 26;19(8):e0305579. doi: 10.1371/journal.pone.0305579 (PMC11346739; doi:10.1371/journal.pone.0305579)
Supplement: S8 Table — The first 71 terms were collected on March 29, 2009, the remaining terms/topics were identified on May 22, 2010. The last 21 terms separated by a horizontal line from the first 140 terms were “Related topics” from Google Trends. (PDF) [file pone.0305579.s011.pdf]

acute bronchitis	body temperature	break a fever	bronchitis
cold or flu	cold vs flu	cough fever	cure the flu
dangerous fever	fever cough	fever flu	fever reducer
flu contagious period	flu contagious	flu duration	flu fever
flu how long	flu in children	flu incubation	flu medicine
flu or cold	flu report	flu test	flu treatment
flu treatments	flu vs cold	get over the flu	high fever
how long is the flu contagious	how long is the flu	how to treat the flu	incubation period for the flu
influenza a and b	influenza a	influenza contagious	influenza incubation period
influenza incubation	influenza symptoms	influenza treatment	influenza type a
is flu contagious	low body	normal body temperature	normal body
over the counter flu	painful cough	pneumonia	reduce a fever
remedies for the flu	robitussin	signs of the flu	sinus infections
sinus	strep	symptoms of bronchitis	symptoms of flu
symptoms of influenza	symptoms of pneumonia	symptoms of the flu	treat flu
treat the flu	treating flu	treating the flu	treatment for flu
treatment for the flu	tussin	tussionex	type a flu
type a influenza	upper respiratory	walking pneumonia	
a influenza	braun thermoscan	chest cold	cold and flu
cold versus flu	contagious flu	cure flu	do i have the flu
ear thermometer	early flu symptoms	expectorant	exposed to flu
fever breaks	fight the flu	flu a symptoms	flu and cold
flu and fever	flu care	flu children	flu complications
flu cough	flu germs	flu headache	flu how long are you contagious
flu incubation period	flu lasts	flu length	flu recovery
flu relief	flu remedies	flu remedy	flu reports
flu symptoms	flu texas	flu versus cold	flu
get rid of the flu	having the flu	how long am i contagious with the flu	how long contagious
how long does flu last	how long does the flu last	how long flu	how long is flu contagious
how to break a fever	how to bring a fever down	how to get rid of the flu	how to treat flu
how to treat the flu at home	human temperature	i have the flu	incubation period for flu
medicine for flu	medicine for the flu	oscillococcinum	over the counter flu medicine
rapid flu	reduce fever	remedies for flu	respiratory flu
signs of flu	strep throat	taking temperature	tessalon
the flu virus	the flu	thermoscan	type a flu symptoms
what to do if you have the flu			
Influenza vaccine	Influenza	Fever	Influenza A virus
Influenza B virus	Common cold	Cough	Sore throat
Virus	Avian influenza	Spanish flu	Headache
Nausea	Flu season	Oseltamivir	Nasal congestion
Canine influenza	Rapid influenza diagnostic test	Theraflu	Dextromethorphan
Rhinorrhea			

Table S8. All search query terms used in this study. The first 71 terms were collected on March 29, 2009, the remaining terms/topics were identified on May 22, 2010. The last 21 terms separated by a horizontal line from the first 140 terms were "Related topics" from Google Trends.
