# Supplementary material for: Incorporating connectivity among Internet search data for enhanced influenza-like illness tracking
Source: PLoS One. 2024 Aug 26;19(8):e0305579. doi: 10.1371/journal.pone.0305579 (PMC11346739; doi:10.1371/journal.pone.0305579)
Supplement: S9 Table — The RMSE, MAE, and correlation measures are reported. The method with the best performance is highlighted in boldface for each metric in each period. (PDF) [file pone.0305579.s012.pdf]

	’09-’23	’09-’15	’09-’10	’10-’11	’11-’12	’12-’13	’13-’14	’14-’15	’15-’16	’16-’17	’17-’18	’18-’19	’19-’20	’20-’21	’21-’22	’22-’23
RMSE																
ARGO-C	**0.307**	**0.349**	0.903	0.174	0.115	**0.253**	**0.214**	**0.321**	**0.183**	**0.214**	**0.256**	**0.180**	**0.432**	0.092	**0.297**	**0.784**
ARGO2	0.316	0.353	0.904	**0.172**	0.104	0.284	0.216	0.330	0.190	0.225	0.273	0.187	0.450	0.096	0.306	0.829
VAR1	0.382	0.409	1.069	0.194	**0.104**	0.372	0.214	0.371	0.212	0.276	0.440	0.271	0.636	**0.067**	0.365	1.011
GFT	–	0.941	**0.466**	0.237	0.245	2.819	0.395	0.418	–	–	–	–	–	–	–	–
naive	0.377	0.406	1.062	0.193	0.105	0.362	0.214	0.360	0.213	0.264	0.436	0.271	0.628	0.068	0.360	0.970
MAE																
ARGO-C	**0.164**	**0.169**	0.486	0.133	0.095	**0.157**	**0.171**	**0.203**	**0.141**	**0.169**	**0.186**	**0.141**	**0.300**	0.069	**0.209**	**0.548**
ARGO2	0.168	0.171	0.487	**0.127**	0.087	0.163	0.178	0.204	0.147	0.173	0.188	0.145	0.306	0.076	0.221	0.597
VAR1	0.198	0.187	0.569	0.147	**0.086**	0.221	0.182	0.213	0.170	0.199	0.323	0.186	0.488	**0.052**	0.282	0.725
GFT	–	0.327	**0.267**	0.195	0.176	1.330	0.334	0.322	–	–	–	–	–	–	–	–
naive	0.198	0.188	0.583	0.143	0.087	0.224	0.180	0.221	0.165	0.193	0.324	0.192	0.497	0.053	0.278	0.690
Correlation																
ARGO-C	**0.968**	**0.934**	0.913	**0.956**	0.630	**0.960**	**0.885**	**0.931**	**0.938**	**0.964**	**0.984**	**0.983**	**0.975**	0.949	**0.931**	**0.943**
ARGO2	0.966	0.933	0.913	0.955	0.690	0.951	0.880	0.929	0.933	0.960	0.983	0.982	0.975	**0.951**	0.923	0.935
VAR1	0.949	0.909	0.876	0.931	0.689	0.915	0.882	0.907	0.921	0.929	0.951	0.961	0.942	0.948	0.888	0.900
GFT	–	0.768	**0.988**	0.911	**0.699**	0.831	0.786	0.917	–	–	–	–	–	–	–	–
naive	0.951	0.914	0.884	0.932	0.693	0.919	0.884	0.914	0.923	0.936	0.951	0.961	0.944	0.947	0.892	0.899

Table S9-1. Comparison of different methods for regional level %ILI estimation in Region 1. The RMSE, MAE, and correlation measures are reported. The method with the best performance is highlighted in boldface for each metric in each period.

	’09-’23	’09-’15	’09-’10	’10-’11	’11-’12	’12-’13	’13-’14	’14-’15	’15-’16	’16-’17	’17-’18	’18-’19	’19-’20	’20-’21	’21-’22	’22-’23
RMSE																
ARGO-C	**0.446**	**0.418**	0.646	**0.385**	0.134	**0.340**	0.337	**0.374**	0.354	**0.540**	0.599	**0.228**	0.953	0.229	**0.552**	**0.885**
ARGO2	0.458	0.430	0.647	0.394	0.125	0.395	**0.330**	0.415	**0.352**	0.577	**0.588**	0.241	**0.929**	0.230	0.579	0.990
VAR1	0.544	0.480	0.793	0.415	**0.120**	0.498	0.366	0.496	0.372	0.639	0.880	0.333	1.250	**0.222**	0.665	1.078
GFT	–	1.037	**0.463**	1.093	0.510	2.370	0.665	0.662	–	–	–	–	–	–	–	–
naive	0.541	0.482	0.799	0.421	0.121	0.498	0.367	0.489	0.381	0.627	0.868	0.333	1.229	0.225	0.667	1.047
MAE																
ARGO-C	**0.280**	**0.268**	0.430	**0.272**	0.110	**0.242**	0.285	**0.314**	**0.292**	**0.442**	**0.429**	**0.186**	**0.567**	**0.174**	**0.412**	**0.626**
ARGO2	0.288	0.275	0.434	0.282	0.101	0.260	**0.283**	0.345	0.296	0.460	0.439	0.193	0.575	0.177	0.417	0.684
VAR1	0.325	0.293	0.450	0.313	**0.097**	0.347	0.308	0.355	0.307	0.462	0.577	0.274	0.896	0.177	0.462	0.822
GFT	–	0.632	**0.364**	0.936	0.440	1.310	0.611	0.487	–	–	–	–	–	–	–	–
naive	0.323	0.297	0.460	0.304	0.097	0.358	0.309	0.360	0.301	0.449	0.572	0.275	0.879	0.180	0.442	0.796
Correlation																
ARGO-C	**0.969**	**0.950**	0.944	**0.938**	0.664	**0.968**	0.848	**0.951**	0.921	**0.946**	0.971	**0.974**	0.950	**0.768**	**0.916**	**0.937**
ARGO2	0.968	0.947	0.946	0.934	**0.693**	0.959	0.849	0.945	**0.922**	0.936	**0.974**	0.971	**0.958**	0.767	0.903	0.921
VAR1	0.952	0.934	0.912	0.927	0.678	0.930	0.831	0.907	0.902	0.912	0.940	0.944	0.901	0.765	0.866	0.878
GFT	–	0.783	**0.981**	0.870	0.398	0.915	**0.871**	0.932	–	–	–	–	–	–	–	–
naive	0.954	0.935	0.914	0.927	0.684	0.931	0.831	0.910	0.902	0.917	0.940	0.945	0.904	0.763	0.867	0.883

Table S9-2. Comparison of different methods for regional level %ILI estimation in Region 2. The RMSE, MAE, and correlation measures are reported. The method with the best performance is highlighted in boldface for each metric in each period.

	’09-’23	’09-’15	’09-’10	’10-’11	’11-’12	’12-’13	’13-’14	’14-’15	’15-’16	’16-’17	’17-’18	’18-’19	’19-’20	’20-’21	’21-’22	’22-’23
RMSE																
ARGO-C	**0.324**	**0.393**	0.755	**0.264**	**0.175**	**0.597**	**0.230**	0.458	**0.219**	**0.367**	**0.379**	**0.194**	**0.295**	0.128	**0.303**	**0.500**
ARGO2	0.350	0.425	0.799	0.291	0.181	0.667	0.243	0.539	0.232	0.403	0.381	0.217	0.342	0.125	0.335	0.564
VAR1	0.460	0.522	0.998	0.327	0.193	0.786	0.311	0.745	0.320	0.530	0.652	0.334	0.708	**0.104**	0.450	0.698
GFT	–	0.859	**0.604**	0.501	0.926	2.188	0.514	**0.380**	–	–	–	–	–	–	–	–
naive	0.457	0.522	0.993	0.337	0.196	0.785	0.312	0.745	0.323	0.506	0.645	0.329	0.699	0.106	0.454	0.673
MAE																
ARGO-C	**0.201**	**0.241**	**0.458**	**0.208**	**0.143**	**0.396**	**0.173**	**0.286**	**0.176**	**0.277**	**0.272**	**0.152**	**0.218**	0.092	**0.209**	**0.361**
ARGO2	0.213	0.255	0.484	0.237	0.143	0.421	0.188	0.339	0.178	0.306	0.293	0.173	0.249	0.097	0.214	0.396
VAR1	0.258	0.282	0.555	0.245	0.153	0.477	0.204	0.410	0.254	0.336	0.407	0.269	0.512	**0.080**	0.284	0.594
GFT	–	0.567	0.474	0.428	0.886	1.760	0.480	0.312	–	–	–	–	–	–	–	–
naive	0.258	0.286	0.555	0.256	0.153	0.482	0.210	0.430	0.260	0.319	0.411	0.262	0.513	0.081	0.281	0.548
Correlation																
ARGO-C	**0.973**	**0.957**	0.960	**0.975**	0.725	**0.916**	**0.975**	0.964	**0.939**	**0.961**	0.984	**0.991**	**0.988**	0.922	**0.954**	**0.952**
ARGO2	0.969	0.951	0.955	0.969	0.712	0.894	0.974	0.954	0.936	0.953	**0.986**	0.989	0.986	**0.932**	0.935	0.938
VAR1	0.944	0.923	0.924	0.950	0.671	0.852	0.924	0.886	0.868	0.905	0.944	0.960	0.924	0.928	0.866	0.891
GFT	–	0.865	**0.991**	0.953	**0.732**	0.891	0.975	**0.985**	–	–	–	–	–	–	–	–
naive	0.946	0.925	0.927	0.947	0.676	0.859	0.926	0.891	0.870	0.916	0.945	0.962	0.927	0.927	0.866	0.892

Table S9-3. Comparison of different methods for regional level %ILI estimation in Region 3. The RMSE, MAE, and correlation measures are reported. The method with the best performance is highlighted in boldface for each metric in each period.

	'09-'23	'09-'15	'09-'10	'10-'11	'11-'12	'12-'13	'13-'14	'14-'15	'15-'16	'16-'17	'17-'18	'18-'19	'19-'20	'20-'21	'21-'22	'22-'23
RMSE																
ARGO-C	**0.335**	**0.356**	0.313	**0.377**	**0.180**	0.524	**0.243**	0.476	**0.179**	**0.390**	0.421	**0.239**	**0.531**	0.139	**0.286**	**0.726**
ARGO2	0.353	0.357	**0.276**	0.394	0.192	**0.524**	0.267	0.517	0.210	0.415	**0.418**	0.300	0.662	0.141	0.321	0.776
VAR1	0.463	0.422	0.286	0.477	0.203	0.568	0.386	0.728	0.281	0.505	0.817	0.483	0.923	**0.098**	0.466	0.994
GFT	–	0.821	0.522	0.908	0.270	2.023	0.407	**0.358**	–	–	–	–	–	–	–	–
naive	0.460	0.423	0.312	0.484	0.205	0.569	0.387	0.734	0.275	0.482	0.809	0.492	0.916	0.100	0.465	0.958
MAE																
ARGO-C	**0.204**	**0.205**	0.225	**0.291**	0.139	**0.325**	**0.166**	0.278	**0.153**	**0.297**	0.284	**0.168**	**0.398**	0.119	**0.206**	**0.456**
ARGO2	0.214	0.206	**0.213**	0.309	**0.138**	0.329	0.172	0.298	0.171	0.327	**0.276**	0.211	0.468	0.126	0.219	0.505
VAR1	0.267	0.234	0.219	0.357	0.158	0.351	0.241	0.416	0.208	0.386	0.529	0.367	0.699	**0.080**	0.308	0.790
GFT	–	0.474	0.462	0.740	0.233	1.468	0.319	**0.255**	–	–	–	–	–	–	–	–
naive	0.268	0.238	0.235	0.365	0.157	0.363	0.244	0.428	0.206	0.368	0.520	0.383	0.695	0.082	0.308	0.751
Correlation																
ARGO-C	**0.977**	**0.963**	0.986	0.966	**0.830**	**0.931**	0.973	0.950	**0.978**	**0.963**	0.987	**0.985**	**0.974**	0.880	**0.973**	**0.919**
ARGO2	0.975	0.962	**0.987**	0.962	0.806	0.923	0.967	0.945	0.974	0.959	**0.988**	0.975	0.958	0.882	0.960	0.907
VAR1	0.957	0.947	0.986	0.945	0.792	0.907	0.920	0.880	0.926	0.931	0.952	0.931	0.908	**0.919**	0.904	0.844
GFT	–	0.835	0.984	**0.975**	0.771	0.892	**0.977**	**0.979**	–	–	–	–	–	–	–	–
naive	0.958	0.948	0.986	0.944	0.793	0.909	0.922	0.881	0.929	0.937	0.951	0.930	0.911	0.916	0.906	0.847

Table S9-4. Comparison of different methods for regional level %ILI estimation in Region 4. The RMSE, MAE, and correlation measures are reported. The method with the best performance is highlighted in boldface for each metric in each period.

	'09-'23	'09-'15	'09-'10	'10-'11	'11-'12	'12-'13	'13-'14	'14-'15	'15-'16	'16-'17	'17-'18	'18-'19	'19-'20	'20-'21	'21-'22	'22-'23
RMSE																
ARGO-C	**0.275**	**0.326**	0.729	**0.241**	**0.162**	**0.378**	**0.197**	0.388	**0.161**	**0.267**	**0.201**	0.233	**0.381**	0.094	**0.247**	**0.534**
ARGO2	0.296	0.347	0.776	0.262	0.176	0.404	0.220	0.425	0.179	0.283	0.219	**0.233**	0.448	0.099	0.259	0.605
VAR1	0.381	0.431	0.932	0.304	0.202	0.491	0.319	0.614	0.271	0.356	0.482	0.301	0.603	**0.066**	0.320	0.765
GFT	–	0.803	**0.480**	0.382	0.528	2.311	0.323	**0.266**	–	–	–	–	–	–	–	–
naive	0.378	0.430	0.917	0.308	0.202	0.487	0.321	0.630	0.271	0.343	0.480	0.301	0.596	0.067	0.317	0.738
MAE																
ARGO-C	**0.159**	**0.178**	**0.410**	**0.183**	**0.134**	**0.243**	**0.121**	0.225	**0.116**	**0.201**	**0.154**	0.169	**0.261**	0.077	**0.179**	**0.410**
ARGO2	0.166	0.185	0.433	0.190	0.141	0.251	0.125	0.258	0.128	0.202	0.166	**0.167**	0.304	0.084	0.187	0.463
VAR1	0.205	0.214	0.514	0.200	0.159	0.292	0.174	0.355	0.209	0.249	0.313	0.234	0.402	**0.056**	0.242	0.604
GFT	–	0.382	0.411	0.315	0.445	1.588	0.227	**0.195**	–	–	–	–	–	–	–	–
naive	0.205	0.218	0.505	0.210	0.157	0.314	0.183	0.367	0.210	0.242	0.323	0.230	0.404	0.056	0.232	0.543
Correlation																
ARGO-C	**0.973**	**0.962**	0.953	**0.973**	**0.891**	**0.951**	0.960	0.954	**0.965**	**0.970**	**0.992**	0.952	**0.968**	0.902	**0.952**	**0.958**
ARGO2	0.970	0.957	0.948	0.966	0.871	0.945	0.950	0.947	0.961	0.968	0.991	**0.953**	0.954	0.902	0.935	0.944
VAR1	0.949	0.933	0.922	0.947	0.835	0.918	0.882	0.882	0.894	0.936	0.952	0.918	0.911	**0.931**	0.879	0.902
GFT	–	0.879	**0.985**	0.938	0.709	0.906	**0.964**	**0.988**	–	–	–	–	–	–	–	–
naive	0.950	0.934	0.924	0.947	0.837	0.920	0.883	0.880	0.897	0.940	0.952	0.920	0.915	0.929	0.881	0.901

Table S9-5. Comparison of different methods for regional level %ILI estimation in Region 5. The RMSE, MAE, and correlation measures are reported. The method with the best performance is highlighted in boldface for each metric in each period.

	'09-'23	'09-'15	'09-'10	'10-'11	'11-'12	'12-'13	'13-'14	'14-'15	'15-'16	'16-'17	'17-'18	'18-'19	'19-'20	'20-'21	'21-'22	'22-'23
RMSE																
ARGO-C	**0.557**	**0.615**	0.893	**0.466**	**0.239**	**0.541**	**0.548**	1.117	0.362	**0.593**	0.803	**0.517**	**1.017**	0.298	0.311	**0.625**
ARGO2	0.578	0.631	**0.884**	0.507	0.273	0.612	0.572	**1.108**	**0.362**	0.647	**0.763**	0.553	1.144	0.314	**0.303**	0.663
VAR1	0.685	0.713	0.977	0.664	0.309	0.759	0.759	1.160	0.441	0.762	1.143	0.795	1.315	**0.249**	0.451	0.785
GFT	–	1.356	0.899	0.639	0.908	3.607	0.770	1.146	–	–	–	–	–	–	–	–
naive	0.678	0.708	1.012	0.665	0.306	0.770	0.747	1.164	0.444	0.727	1.131	0.791	1.300	0.251	0.456	0.753
MAE																
ARGO-C	**0.338**	**0.370**	0.593	**0.366**	**0.175**	**0.407**	**0.361**	**0.627**	0.300	**0.399**	**0.424**	**0.353**	**0.707**	0.240	0.222	**0.499**
ARGO2	0.352	0.385	**0.579**	0.402	0.188	0.442	0.368	0.678	**0.293**	0.417	0.441	0.373	0.788	0.250	**0.220**	0.542
VAR1	0.410	0.421	0.580	0.477	0.212	0.506	0.493	0.732	0.364	0.555	0.759	0.608	0.978	**0.164**	0.304	0.648
GFT	–	0.714	0.618	0.439	0.848	2.567	0.544	0.661	–	–	–	–	–	–	–	–
naive	0.411	0.424	0.625	0.461	0.207	0.534	0.498	0.752	0.359	0.544	0.750	0.618	0.983	0.168	0.323	0.624
Correlation																
ARGO-C	**0.973**	**0.959**	0.961	0.974	0.909	**0.967**	**0.963**	0.896	**0.890**	**0.972**	0.981	**0.984**	**0.954**	**0.691**	**0.949**	**0.952**
ARGO2	0.971	0.958	0.962	0.969	0.879	0.958	0.959	0.899	0.886	0.968	**0.982**	0.980	0.942	0.675	0.946	0.947
VAR1	0.958	0.945	0.952	0.943	0.850	0.934	0.924	0.890	0.833	0.944	0.957	0.947	0.916	0.684	0.878	0.911
GFT	–	0.878	**0.980**	**0.978**	**0.914**	0.943	0.956	**0.928**	–	–	–	–	–	–	–	–
naive	0.960	0.947	0.954	0.944	0.855	0.933	0.927	0.893	0.837	0.950	0.957	0.949	0.920	0.681	0.877	0.916

Table S9-6. Comparison of different methods for regional level %ILI estimation in Region 6. The RMSE, MAE, and correlation measures are reported. The method with the best performance is highlighted in boldface for each metric in each period.

	’09-’23	’09-’15	’09-’10	’10-’11	’11-’12	’12-’13	’13-’14	’14-’15	’15-’16	’16-’17	’17-’18	’18-’19	’19-’20	’20-’21	’21-’22	’22-’23
RMSE																
ARGO-C	**0.423**	**0.463**	**0.921**	**0.387**	**0.298**	**0.470**	**0.263**	**0.377**	**0.211**	**0.448**	**0.461**	**0.487**	**0.584**	0.260	0.268	**0.781**
ARGO2	0.453	0.493	0.969	0.402	0.324	0.535	0.309	0.459	0.212	0.489	0.510	0.515	0.709	0.266	**0.255**	0.840
VAR1	0.552	0.572	1.122	0.491	0.348	0.631	0.459	0.621	0.245	0.583	0.908	0.656	0.917	**0.231**	0.305	1.006
GFT	–	0.917	1.968	1.077	0.603	0.718	0.322	0.856	–	–	–	–	–	–	–	–
naive	0.546	0.569	1.111	0.495	0.343	0.637	0.468	0.623	0.246	0.555	0.903	0.651	0.903	0.236	0.312	0.964
MAE																
ARGO-C	**0.244**	**0.261**	**0.600**	**0.302**	**0.229**	**0.290**	**0.179**	**0.242**	0.166	**0.300**	**0.314**	**0.357**	**0.405**	0.140	0.205	**0.481**
ARGO2	0.260	0.280	0.641	0.312	0.242	0.313	0.203	0.302	**0.164**	0.319	0.342	0.375	0.488	0.157	**0.186**	0.516
VAR1	0.300	0.304	0.698	0.346	0.262	0.365	0.278	0.376	0.200	0.404	0.595	0.481	0.630	**0.116**	0.214	0.705
GFT	–	0.506	1.385	0.791	0.436	0.539	0.200	0.493	–	–	–	–	–	–	–	–
naive	0.303	0.313	0.716	0.354	0.259	0.390	0.298	0.412	0.195	0.397	0.600	0.467	0.630	0.119	0.224	0.658
Correlation																
ARGO-C	**0.969**	**0.959**	0.943	0.953	0.941	**0.961**	**0.970**	**0.975**	0.901	**0.961**	**0.988**	**0.958**	**0.970**	0.798	**0.963**	**0.931**
ARGO2	0.965	0.955	0.938	0.949	0.931	0.952	0.960	0.967	**0.903**	0.954	0.986	0.953	0.955	0.792	0.956	0.918
VAR1	0.946	0.936	0.913	0.916	0.917	0.929	0.899	0.921	0.855	0.930	0.951	0.913	0.924	**0.817**	0.928	0.876
GFT	–	0.846	**0.985**	**0.960**	**0.953**	0.934	0.964	0.963	–	–	–	–	–	–	–	–
naive	0.948	0.938	0.918	0.917	0.919	0.929	0.898	0.919	0.858	0.936	0.952	0.917	0.928	0.813	0.924	0.876

Table S9-7. Comparison of different methods for regional level %ILI estimation in Region 7. The RMSE, MAE, and correlation measures are reported. The method with the best performance is highlighted in boldface for each metric in each period.

	’09-’23	’09-’15	’09-’10	’10-’11	’11-’12	’12-’13	’13-’14	’14-’15	’15-’16	’16-’17	’17-’18	’18-’19	’19-’20	’20-’21	’21-’22	’22-’23
RMSE																
ARGO-C	**0.307**	**0.336**	**0.812**	0.237	**0.152**	**0.213**	0.283	**0.294**	**0.132**	0.351	**0.169**	**0.470**	**0.361**	0.134	**0.244**	**0.710**
ARGO2	0.319	0.347	0.831	**0.219**	0.169	0.252	0.300	0.323	0.151	**0.337**	0.172	0.476	0.415	0.139	0.259	0.758
VAR1	0.392	0.410	0.985	0.226	0.180	0.341	0.369	0.389	0.189	0.363	0.260	0.562	0.727	**0.088**	0.317	0.919
GFT	–	0.622	1.502	0.382	0.387	0.912	**0.282**	0.455	–	–	–	–	–	–	–	–
naive	0.385	0.402	0.969	0.229	0.177	0.341	0.374	0.388	0.186	0.369	0.250	0.532	0.721	0.088	0.315	0.894
MAE																
ARGO-C	**0.177**	**0.172**	**0.403**	0.189	**0.105**	**0.161**	**0.169**	**0.199**	**0.109**	0.262	**0.130**	**0.372**	**0.262**	0.105	**0.187**	**0.554**
ARGO2	0.182	0.177	0.411	0.181	0.117	0.170	0.177	0.225	0.121	**0.250**	0.132	0.376	0.290	0.114	0.195	0.595
VAR1	0.212	0.196	0.496	**0.156**	0.130	0.234	0.217	0.227	0.150	0.269	0.193	0.427	0.516	**0.072**	0.242	0.734
GFT	–	0.315	0.863	0.273	0.339	0.506	0.234	0.289	–	–	–	–	–	–	–	–
naive	0.211	0.199	0.512	0.162	0.124	0.241	0.226	0.239	0.147	0.273	0.179	0.397	0.513	0.072	0.236	0.720
Correlation																
ARGO-C	**0.976**	**0.959**	0.947	0.952	**0.905**	**0.983**	0.921	**0.968**	**0.969**	0.824	0.986	**0.952**	**0.985**	0.871	**0.939**	**0.957**
ARGO2	0.974	0.957	0.945	**0.961**	0.885	0.978	0.910	0.964	0.960	**0.833**	**0.986**	0.950	0.979	0.870	0.921	0.950
VAR1	0.960	0.938	0.923	0.941	0.874	0.955	0.866	0.930	0.931	0.797	0.963	0.924	0.933	**0.891**	0.848	0.917
GFT	–	0.859	**0.988**	0.929	0.872	0.926	**0.955**	0.930	–	–	–	–	–	–	–	–
naive	0.961	0.941	0.927	0.941	0.877	0.955	0.867	0.931	0.935	0.803	0.963	0.930	0.935	0.889	0.848	0.915

Table S9-8. Comparison of different methods for regional level %ILI estimation in Region 8. The RMSE, MAE, and correlation measures are reported. The method with the best performance is highlighted in boldface for each metric in each period.

	’09-’23	’09-’15	’09-’10	’10-’11	’11-’12	’12-’13	’13-’14	’14-’15	’15-’16	’16-’17	’17-’18	’18-’19	’19-’20	’20-’21	’21-’22	’22-’23
RMSE																
ARGO-C	**0.347**	**0.437**	0.665	0.358	**0.382**	**0.474**	**0.297**	**0.284**	**0.224**	0.199	**0.404**	0.181	0.306	0.135	**0.229**	**0.639**
ARGO2	0.359	0.453	**0.654**	**0.353**	0.394	0.525	0.319	0.330	0.244	**0.186**	0.413	**0.178**	**0.288**	**0.134**	0.250	0.700
VAR1	0.406	0.470	0.664	0.395	0.419	0.538	0.382	0.380	0.315	0.208	0.622	0.224	0.462	0.137	0.353	0.877
GFT	–	1.091	1.101	1.168	0.567	2.536	0.593	0.540	–	–	–	–	–	–	–	–
naive	0.405	0.475	0.676	0.382	0.438	0.544	0.385	0.382	0.312	0.210	0.606	0.222	0.461	0.139	0.358	0.828
MAE																
ARGO-C	**0.213**	**0.275**	0.502	**0.261**	**0.308**	**0.325**	**0.204**	**0.201**	**0.172**	**0.142**	**0.265**	0.132	0.230	0.109	**0.183**	**0.453**
ARGO2	0.217	0.282	**0.488**	0.270	0.321	0.339	0.214	0.219	0.186	0.149	0.266	**0.127**	**0.209**	0.109	0.184	0.480
VAR1	0.247	0.297	0.510	0.320	0.349	0.327	0.247	0.258	0.243	0.161	0.357	0.181	0.338	**0.106**	0.214	0.726
GFT	–	0.690	0.995	1.090	0.500	1.296	0.393	0.410	–	–	–	–	–	–	–	–
naive	0.247	0.299	0.514	0.298	0.368	0.354	0.253	0.260	0.244	0.167	0.365	0.182	0.333	0.107	0.221	0.656
Correlation																
ARGO-C	**0.956**	**0.921**	0.891	**0.944**	0.768	**0.922**	**0.963**	**0.967**	**0.965**	0.945	**0.959**	0.972	0.971	0.948	**0.957**	**0.959**
ARGO2	0.953	0.915	0.893	0.944	0.751	0.902	0.958	0.960	0.960	**0.946**	0.955	**0.974**	**0.974**	**0.949**	0.946	0.947
VAR1	0.940	0.909	0.887	0.922	0.728	0.894	0.925	0.930	0.930	0.933	0.897	0.957	0.929	0.943	0.887	0.895
GFT	–	0.806	**0.945**	0.905	**0.844**	0.900	0.937	0.917	–	–	–	–	–	–	–	–
naive	0.941	0.910	0.890	0.924	0.728	0.894	0.926	0.931	0.933	0.933	0.901	0.958	0.930	0.942	0.886	0.896

Table S9-9. Comparison of different methods for regional level %ILI estimation in Region 9. The RMSE, MAE, and correlation measures are reported.. The method with the best performance is highlighted in boldface for each metric in each period.

	'09-'23	'09-'15	'09-'10	'10-'11	'11-'12	'12-'13	'13-'14	'14-'15	'15-'16	'16-'17	'17-'18	'18-'19	'19-'20	'20-'21	'21-'22	'22-'23
RMSE																
ARGO-C	**0.383**	**0.419**	0.697	0.466	0.225	**0.379**	**0.376**	**0.259**	**0.254**	**0.438**	**0.345**	**0.282**	**0.453**	0.122	0.205	**1.144**
ARGO2	0.396	0.428	**0.687**	0.469	**0.221**	0.406	0.399	0.267	0.259	0.452	0.350	0.308	0.530	0.120	**0.179**	1.194
VAR1	0.471	0.469	0.733	0.477	0.235	0.438	0.463	0.330	0.277	0.493	0.504	0.410	0.774	**0.085**	0.267	1.526
GFT	–	1.083	0.800	**0.423**	0.629	2.880	0.869	0.339	–	–	–	–	–	–	–	–
naive	0.457	0.457	0.705	0.493	0.237	0.451	0.467	0.325	0.271	0.506	0.505	0.400	0.783	0.085	0.259	1.393
MAE																
ARGO-C	**0.223**	**0.253**	0.513	0.340	**0.174**	**0.254**	**0.221**	**0.177**	**0.201**	**0.282**	0.241	**0.208**	**0.340**	0.094	0.170	**0.751**
ARGO2	0.225	0.256	0.494	0.325	0.179	0.260	0.226	0.187	0.206	0.282	**0.220**	0.232	0.380	0.091	**0.144**	0.759
VAR1	0.253	0.260	**0.454**	**0.306**	0.202	0.263	0.270	0.245	0.213	0.282	0.292	0.282	0.569	**0.063**	0.185	1.184
GFT	–	0.644	0.610	0.355	0.597	2.175	0.764	0.254	–	–	–	–	–	–	–	–
naive	0.255	0.270	0.474	0.335	0.208	0.286	0.280	0.248	0.208	0.330	0.321	0.277	0.564	0.064	0.176	0.997
Correlation																
ARGO-C	**0.958**	**0.944**	0.964	0.869	0.852	**0.920**	0.926	0.959	**0.903**	**0.880**	0.962	**0.968**	**0.969**	0.900	0.932	**0.937**
ARGO2	0.956	0.942	0.964	0.866	0.860	0.905	0.916	**0.960**	0.899	0.872	**0.962**	0.962	0.958	0.906	**0.946**	0.930
VAR1	0.936	0.931	0.956	0.859	0.847	0.890	0.882	0.933	0.885	0.849	0.919	0.927	0.899	**0.927**	0.877	0.891
GFT	–	0.804	**0.985**	**0.919**	**0.887**	0.911	**0.974**	0.933	–	–	–	–	–	–	–	–
naive	0.942	0.935	0.962	0.859	0.850	0.887	0.883	0.933	0.892	0.850	0.919	0.932	0.898	0.925	0.882	0.891

Table S9-10. Comparison of different methods for regional level %ILI estimation in Region 10. The RMSE, MAE, and correlation measures are reported. The method with the best performance is highlighted in boldface for each metric in each period.
