# Supplementary material for: Incorporating connectivity among Internet search data for enhanced influenza-like illness tracking
Source: PLoS One. 2024 Aug 26;19(8):e0305579. doi: 10.1371/journal.pone.0305579 (PMC11346739; doi:10.1371/journal.pone.0305579)
Supplement: S10 Table — The MSE, MAE, and correlation are reported. The method with the best performance is highlighted in boldface for each metric in each period. (PDF) [file pone.0305579.s013.pdf]

	Whole period	'14-'23	post-COVID	GFT period	'14-'15	'15-'16	'16-'17	'17-'18	'18-'19	'19-'20	'20-'21	'21-'22	'22-'23
RMSE													
ARGO-C	**0.831**	**0.752**	**0.577**	**1.756**	**1.922**	0.625	**0.544**	**0.846**	**0.504**	**0.698**	0.198	**0.274**	**1.507**
ARGOX	0.873	0.797	0.632	1.865	2.045	0.607	0.596	0.864	0.530	0.779	0.192	0.289	1.653
VAR1	2.407	2.383	2.336	4.624	5.083	1.324	2.231	3.394	1.693	3.559	0.351	0.687	5.138
GFT	–	–	–	2.891	3.183	–	–	–	–	–	–	–	–
naive	1.115	0.977	0.646	2.271	2.495	**0.591**	0.831	1.315	0.785	1.043	**0.142**	0.453	1.645
MAE													
ARGO-C	**0.462**	**0.400**	**0.284**	**1.107**	**1.262**	0.480	**0.407**	**0.459**	**0.342**	**0.492**	0.155	0.213	**1.067**
ARGOX	0.477	0.413	0.294	1.119	1.292	0.463	0.467	0.482	0.369	0.530	0.154	**0.196**	1.210
VAR1	1.349	1.271	1.125	2.847	3.324	1.156	1.665	2.169	1.072	2.374	0.293	0.599	4.203
GFT	–	–	–	1.934	2.321	–	–	–	–	–	–	–	–
naive	0.595	0.501	0.325	1.316	1.552	**0.444**	0.639	0.757	0.573	0.782	**0.121**	0.307	1.189
Correlation													
ARGO-C	**0.956**	**0.956**	**0.945**	0.911	0.894	0.815	**0.958**	**0.976**	**0.970**	**0.962**	0.709	**0.980**	**0.832**
ARGOX	0.952	0.951	0.936	0.898	0.878	0.831	0.950	0.976	0.966	0.951	0.721	0.970	0.799
VAR1	0.675	0.517	0.632	0.715	0.655	0.820	0.639	0.615	0.859	0.278	-0.105	0.777	-0.040
GFT	–	–	–	**0.960**	**0.952**	–	–	–	–	–	–	–	–
naive	0.923	0.928	0.933	0.855	0.826	**0.851**	0.906	0.941	0.925	0.908	**0.795**	0.906	0.808

Table S10-1. Comparison of different methods for state-level %ILI estimation in Alabama. The MSE, MAE, and correlation are reported. The method with the best performance is highlighted in boldface for each metric in each period.

	Whole period	'14-'23	post-COVID	GFT period	'14-'15	'15-'16	'16-'17	'17-'18	'18-'19	'19-'20	'20-'21	'21-'22	'22-'23
RMSE													
ARGO-C	**0.961**	**0.849**	**0.586**	0.663	0.630	**0.828**	**0.705**	**0.634**	**1.097**	1.962	0.301	**0.631**	**0.625**
ARGOX	0.966	0.855	0.596	0.654	0.622	0.832	0.707	0.635	1.108	1.979	0.298	0.655	0.630
VAR1	1.058	0.945	0.690	0.689	0.656	1.159	0.929	1.095	1.215	**1.847**	0.305	0.789	1.071
GFT	–	–	–	1.015	1.042	–	–	–	–	–	–	–	–
naive	1.002	0.896	0.654	**0.646**	**0.605**	0.843	0.783	0.645	1.126	2.047	**0.287**	0.788	0.660
MAE													
ARGO-C	**0.594**	**0.519**	**0.381**	0.493	0.460	**0.565**	**0.519**	**0.508**	**0.765**	1.267	0.208	0.363	0.437
ARGOX	0.595	0.521	0.382	0.488	0.454	0.569	0.524	0.515	0.774	1.265	**0.200**	**0.361**	**0.436**
VAR1	0.684	0.601	0.449	0.538	0.502	0.749	0.686	0.938	0.836	**1.148**	0.214	0.447	0.860
GFT	–	–	–	0.867	0.877	–	–	–	–	–	–	–	–
naive	0.619	0.552	0.429	**0.479**	**0.438**	0.596	0.604	0.519	0.821	1.259	0.210	0.479	0.485
Correlation													
ARGO-C	**0.871**	**0.873**	**0.821**	0.731	0.777	0.818	**0.796**	**0.915**	**0.881**	**0.629**	0.604	**0.795**	**0.838**
ARGOX	0.870	0.871	0.808	0.737	0.782	0.816	0.796	0.914	0.879	0.620	**0.605**	0.782	0.828
VAR1	0.856	0.851	0.701	0.756	0.807	0.802	0.743	0.893	0.870	0.614	0.552	0.741	0.678
GFT	–	–	–	0.623	0.776	–	–	–	–	–	–	–	–
naive	0.864	0.864	0.774	**0.768**	**0.812**	**0.820**	0.769	0.907	0.880	0.603	0.533	0.712	0.759

Table S10-2. Comparison of different methods for state-level %ILI estimation in Alaska. The MSE, MAE, and correlation are reported. The method with the best performance is highlighted in boldface for each metric in each period.

	Whole period	'14-'23	post-COVID	GFT period	'14-'15	'15-'16	'16-'17	'17-'18	'18-'19	'19-'20	'20-'21	'21-'22	'22-'23
RMSE													
ARGO-C	**0.396**	**0.378**	0.342	**0.246**	**0.256**	**0.391**	0.319	**0.587**	0.315	0.662	0.272	0.336	**0.483**
ARGOX	0.404	0.382	**0.338**	0.253	0.266	0.413	0.311	0.645	**0.309**	**0.629**	**0.259**	**0.335**	0.513
VAR1	2.372	2.080	1.384	4.487	4.938	1.218	2.423	2.891	1.615	2.398	0.365	0.539	3.543
GFT	–	–	–	1.160	1.253	–	–	–	–	–	–	–	–
naive	0.454	0.432	0.387	0.280	0.298	0.401	**0.304**	0.787	0.363	0.707	0.279	0.424	0.679
MAE													
ARGO-C	**0.290**	**0.277**	0.254	0.187	0.193	**0.286**	0.267	**0.448**	0.267	0.533	0.227	**0.243**	**0.370**
ARGOX	0.292	0.278	**0.251**	**0.182**	**0.192**	0.304	0.259	0.484	**0.266**	**0.505**	**0.212**	0.246	0.401
VAR1	1.409	1.164	0.708	2.910	3.479	0.976	1.960	1.892	0.976	1.839	0.316	0.343	2.430
GFT	–	–	–	1.010	1.131	–	–	–	–	–	–	–	–
naive	0.309	0.294	0.266	0.216	0.233	0.293	**0.254**	0.539	0.269	0.542	0.217	0.292	0.525
Correlation													
ARGO-C	**0.953**	**0.952**	0.938	**0.959**	**0.955**	**0.945**	0.862	**0.942**	0.921	0.846	0.770	**0.888**	**0.954**
ARGOX	0.951	0.951	**0.940**	0.958	0.953	0.938	0.864	0.928	**0.925**	**0.855**	**0.780**	0.882	0.945
VAR1	0.613	0.660	0.754	0.725	0.666	0.468	0.680	0.719	0.707	0.799	0.516	0.861	0.484
GFT	–	–	–	0.923	0.912	–	–	–	–	–	–	–	–
naive	0.939	0.939	0.924	0.941	0.932	0.939	**0.865**	0.884	0.901	0.816	0.749	0.816	0.904

Table S10-3. Comparison of different methods for state-level %ILI estimation in Arizona. The MSE, MAE, and correlation are reported. The method with the best performance is highlighted in boldface for each metric in each period.

	Whole period	’14-’23	post-COVID	GFT period	’14-’15	’15-’16	’16-’17	’17-’18	’18-’19	’19-’20	’20-’21	’21-’22	’22-’23
RMSE													
ARGO-C	**0.821**	**0.741**	**0.564**	**1.050**	**1.144**	0.622	**0.842**	**0.905**	0.830	**1.576**	0.195	0.405	**0.718**
ARGOX	0.853	0.780	0.622	1.102	1.201	**0.612**	0.897	0.973	**0.805**	1.716	0.199	**0.382**	0.798
VAR1	1.103	0.950	0.565	1.382	1.514	0.656	1.154	1.597	1.041	1.812	**0.167**	0.417	0.885
GFT	–	–	–	1.391	1.466	–	–	–	–	–	–	–	–
naive	1.049	0.919	0.609	1.328	1.453	0.647	1.080	1.444	0.987	1.871	0.168	0.421	0.846
MAE													
ARGO-C	**0.518**	**0.450**	0.324	**0.601**	**0.657**	0.456	**0.596**	**0.698**	**0.569**	**1.159**	0.155	0.292	**0.579**
ARGOX	0.530	0.463	0.339	0.649	0.716	**0.446**	0.605	0.732	0.584	1.228	0.159	**0.274**	0.672
VAR1	0.636	0.521	**0.306**	0.809	0.923	0.464	0.713	1.012	0.771	1.202	0.142	0.309	0.729
GFT	–	–	–	1.179	1.237	–	–	–	–	–	–	–	–
naive	0.627	0.518	0.316	0.747	0.847	0.472	0.708	1.042	0.729	1.253	**0.136**	0.326	0.704
Correlation													
ARGO-C	**0.946**	**0.943**	**0.923**	0.922	0.907	0.865	**0.937**	**0.975**	0.938	**0.826**	0.722	**0.859**	**0.927**
ARGOX	0.942	0.937	0.910	0.913	0.896	**0.872**	0.928	0.969	**0.940**	0.795	**0.723**	0.856	0.910
VAR1	0.911	0.908	0.914	0.877	0.853	0.860	0.885	0.914	0.899	0.753	0.712	0.814	0.892
GFT	–	–	–	**0.971**	**0.967**	–	–	–	–	–	–	–	–
naive	0.913	0.914	0.911	0.878	0.854	0.866	0.893	0.918	0.903	0.755	0.711	0.812	0.900

Table S10-4. Comparison of different methods for state-level %ILI estimation in Arkansas. The MSE, MAE, and correlation are reported. The method with the best performance is highlighted in boldface for each metric in each period.

	Whole period	’14-’23	post-COVID	GFT period	’14-’15	’15-’16	’16-’17	’17-’18	’18-’19	’19-’20	’20-’21	’21-’22	’22-’23
RMSE													
ARGO-C	**0.252**	**0.267**	**0.292**	**0.255**	**0.275**	**0.278**	0.247	**0.389**	0.235	0.342	0.142	**0.248**	**0.649**
ARGOX	0.260	0.279	0.312	0.276	0.298	0.303	**0.243**	0.416	**0.217**	**0.326**	**0.133**	0.261	0.738
VAR1	0.793	0.872	1.003	1.287	1.347	0.648	0.640	0.987	0.595	0.810	0.264	0.671	2.477
GFT	–	–	–	0.535	0.555	–	–	–	–	–	–	–	–
naive	0.344	0.372	0.419	0.370	0.402	0.354	0.252	0.640	0.262	0.492	0.150	0.410	0.937
MAE													
ARGO-C	0.182	**0.185**	**0.192**	**0.197**	**0.216**	**0.219**	**0.179**	**0.263**	0.188	0.251	0.115	**0.206**	**0.513**
ARGOX	**0.181**	0.186	0.194	0.205	0.225	0.241	0.183	0.274	**0.165**	**0.239**	**0.109**	0.208	0.559
VAR1	0.636	0.646	0.665	1.206	1.264	0.472	0.570	0.836	0.475	0.620	0.206	0.513	2.311
GFT	–	–	–	0.398	0.400	–	–	–	–	–	–	–	–
naive	0.220	0.228	0.244	0.247	0.275	0.277	0.194	0.390	0.220	0.347	0.119	0.243	0.739
Correlation													
ARGO-C	**0.974**	**0.977**	**0.981**	**0.967**	**0.961**	**0.942**	**0.934**	**0.959**	0.950	0.970	0.947	**0.955**	**0.953**
ARGOX	0.972	0.975	0.978	0.963	0.956	0.930	0.930	0.953	**0.959**	**0.973**	**0.952**	0.948	0.933
VAR1	0.774	0.750	0.812	0.902	0.883	0.879	0.482	0.902	0.853	0.836	0.807	0.597	0.900
GFT	–	–	–	0.941	0.936	–	–	–	–	–	–	–	–
naive	0.952	0.956	0.960	0.928	0.914	0.907	0.917	0.889	0.940	0.934	0.940	0.873	0.890

Table S10-5. Comparison of different methods for state-level %ILI estimation in California. The MSE, MAE, and correlation are reported. The method with the best performance is highlighted in boldface for each metric in each period.

	Whole period	’14-’23	post-COVID	GFT period	’14-’15	’15-’16	’16-’17	’17-’18	’18-’19	’19-’20	’20-’21	’21-’22	’22-’23
RMSE													
ARGO-C	0.422	**0.410**	**0.386**	0.410	0.450	**0.151**	0.608	0.225	0.790	**0.422**	0.150	**0.370**	**0.862**
ARGOX	**0.417**	0.411	0.401	**0.402**	**0.441**	0.155	**0.589**	**0.207**	0.783	0.443	0.146	0.384	0.906
VAR1	1.414	1.289	1.019	1.671	1.669	1.512	0.880	1.602	1.921	1.829	0.621	1.340	1.582
GFT	–	–	–	0.809	0.886	–	–	–	–	–	–	–	–
naive	0.461	0.489	0.536	0.478	0.525	0.208	0.685	0.270	**0.725**	0.829	**0.130**	0.376	1.143
MAE													
ARGO-C	0.272	0.262	**0.244**	**0.242**	**0.279**	0.117	0.445	0.180	0.629	**0.305**	0.117	0.281	**0.673**
ARGOX	**0.270**	**0.262**	0.248	0.245	0.282	**0.113**	**0.436**	**0.156**	0.621	0.332	0.114	**0.276**	0.706
VAR1	1.202	1.039	0.735	1.611	1.599	1.438	0.771	1.368	1.624	1.488	0.429	1.162	1.242
GFT	–	–	–	0.458	0.514	–	–	–	–	–	–	–	–
naive	0.303	0.305	0.309	0.304	0.352	0.166	0.506	0.216	**0.562**	0.596	**0.100**	0.303	0.915
Correlation													
ARGO-C	0.970	**0.975**	**0.981**	0.930	0.921	**0.939**	0.450	0.977	**0.913**	**0.983**	0.866	**0.918**	**0.955**
ARGOX	**0.971**	0.974	0.979	**0.933**	**0.924**	0.935	0.481	**0.980**	0.913	0.982	**0.875**	0.913	0.949
VAR1	0.630	0.725	0.864	0.854	0.832	0.865	**0.626**	0.790	0.846	0.872	-0.210	0.189	0.849
GFT	–	–	–	0.841	0.816	–	–	–	–	–	–	–	–
naive	0.964	0.964	0.964	0.902	0.886	0.885	0.374	0.959	0.904	0.930	0.854	0.903	0.917

Table S10-6. Comparison of different methods for state-level %ILI estimation in Colorado. The MSE, MAE, and correlation are reported. The method with the best performance is highlighted in boldface for each metric in each period.

	Whole period	'14-'23	post-COVID	GFT period	'14-'15	'15-'16	'16-'17	'17-'18	'18-'19	'19-'20	'20-'21	'21-'22	'22-'23
RMSE													
ARGO-C	**0.534**	**0.489**	**0.393**	0.677	0.743	0.471	0.409	0.462	0.445	**0.724**	0.162	**0.337**	**0.734**
ARGOX	0.536	0.493	0.400	0.680	0.746	0.471	**0.395**	**0.447**	**0.412**	0.769	0.158	0.371	0.762
VAR1	1.425	1.274	0.933	1.684	1.840	1.548	1.589	2.358	0.828	1.163	0.267	1.012	2.018
GFT	–	–	–	1.475	1.614	–	–	–	–	–	–	–	–
naive	0.576	0.537	0.456	**0.625**	**0.683**	**0.462**	0.492	0.575	0.493	0.865	**0.149**	0.414	0.935
MAE													
ARGO-C	0.365	0.316	**0.226**	0.440	0.514	0.376	0.314	0.351	0.352	0.543	0.127	**0.211**	**0.520**
ARGOX	**0.358**	**0.313**	0.231	0.433	0.504	0.382	**0.300**	**0.336**	**0.311**	**0.540**	0.120	0.221	0.575
VAR1	1.078	0.907	0.589	1.311	1.496	1.363	1.253	2.055	0.614	1.020	0.220	0.865	1.595
GFT	–	–	–	1.092	1.254	–	–	–	–	–	–	–	–
naive	0.376	0.336	0.261	**0.348**	**0.388**	**0.360**	0.379	0.423	0.367	0.640	**0.110**	0.281	0.741
Correlation													
ARGO-C	0.959	**0.963**	**0.972**	0.901	0.874	0.884	0.948	0.966	0.946	**0.967**	**0.904**	**0.937**	**0.935**
ARGOX	**0.959**	0.963	0.970	0.901	0.875	0.881	**0.952**	**0.968**	**0.954**	0.965	0.903	0.913	0.928
VAR1	0.729	0.755	0.907	0.642	0.564	0.697	0.679	0.950	0.840	0.902	0.598	0.697	0.904
GFT	–	–	–	0.748	0.686	–	–	–	–	–	–	–	–
naive	0.952	0.956	0.963	**0.914**	**0.894**	**0.887**	0.925	0.948	0.934	0.945	0.898	0.850	0.889

Table S10-7. Comparison of different methods for state-level %ILI estimation in Connecticut. The MSE, MAE, and correlation are reported. The method with the best performance is highlighted in boldface for each metric in each period.

	Whole period	'14-'23	post-COVID	GFT period	'14-'15	'15-'16	'16-'17	'17-'18	'18-'19	'19-'20	'20-'21	'21-'22	'22-'23
RMSE													
ARGO-C	**0.421**	**0.404**	**0.370**	0.549	0.604	0.252	0.203	0.689	0.256	**0.808**	0.190	**0.293**	**0.315**
ARGOX	0.421	0.405	0.373	0.553	0.608	**0.243**	**0.186**	**0.684**	**0.254**	0.836	**0.186**	0.295	0.324
VAR1	1.898	1.755	1.453	1.747	1.882	1.808	2.227	2.454	2.420	3.279	0.426	0.696	2.519
GFT	–	–	–	2.085	2.232	–	–	–	–	–	–	–	–
naive	0.453	0.453	0.452	**0.509**	**0.560**	0.256	0.230	0.785	0.293	0.954	0.202	0.376	0.502
MAE													
ARGO-C	0.217	**0.218**	**0.221**	0.272	0.320	0.189	0.159	0.372	**0.196**	**0.547**	0.153	**0.229**	0.254
ARGOX	**0.217**	0.218	0.222	0.280	0.330	0.184	**0.147**	**0.354**	0.201	0.561	**0.151**	0.235	**0.254**
VAR1	1.529	1.302	0.880	1.504	1.644	1.659	2.022	2.240	2.300	2.877	0.374	0.586	2.096
GFT	–	–	–	1.880	2.032	–	–	–	–	–	–	–	–
naive	0.226	0.233	0.247	**0.245**	**0.288**	**0.173**	0.173	0.468	0.208	0.618	0.163	0.277	0.396
Correlation													
ARGO-C	**0.873**	**0.887**	**0.896**	0.825	0.811	0.829	0.767	0.883	0.907	**0.809**	0.661	**0.897**	**0.935**
ARGOX	0.873	0.886	0.894	0.823	0.809	**0.840**	0.810	**0.885**	**0.912**	0.800	0.671	0.883	0.932
VAR1	0.708	0.671	0.709	0.449	0.380	0.494	**0.835**	0.781	0.775	0.609	**0.735**	0.845	0.875
GFT	–	–	–	0.737	0.714	–	–	–	–	–	–	–	–
naive	0.859	0.864	0.846	**0.859**	**0.847**	0.832	0.739	0.848	0.882	0.735	0.638	0.809	0.844

Table S10-8. Comparison of different methods for state-level %ILI estimation in Delaware. The MSE, MAE, and correlation are reported. The method with the best performance is highlighted in boldface for each metric in each period.

	Whole period	'14-'23	post-COVID	GFT period	'14-'15	'15-'16	'16-'17	'17-'18	'18-'19	'19-'20	'20-'21	'21-'22	'22-'23
RMSE													
ARGO-C	1.421	1.264	0.904	1.515	1.596	**1.595**	**2.054**	1.190	0.494	0.802	0.508	0.728	1.660
ARGOX	**1.418**	**1.262**	0.902	**1.509**	**1.587**	1.615	2.062	1.226	**0.459**	0.798	0.512	**0.714**	1.673
VAR1	1.523	1.346	0.934	1.560	1.651	1.632	2.157	1.604	0.542	0.861	**0.459**	0.777	1.601
GFT	–	–	–	5.236	5.015	–	–	–	–	–	–	–	–
naive	1.447	1.276	**0.872**	1.588	1.676	1.669	2.193	**0.846**	0.529	**0.770**	0.496	0.796	**1.465**
MAE													
ARGO-C	0.977	0.860	0.645	1.153	1.200	1.329	**1.422**	0.956	0.348	0.647	0.352	0.561	1.409
ARGOX	0.978	0.863	0.648	**1.148**	**1.198**	1.295	1.447	1.018	**0.315**	0.642	0.380	**0.546**	1.432
VAR1	1.069	0.925	0.657	1.220	1.289	**1.265**	1.505	1.483	0.438	0.638	0.338	0.588	1.313
GFT	–	–	–	4.935	4.686	–	–	–	–	–	–	–	–
naive	**0.890**	**0.788**	**0.601**	1.274	1.342	1.277	1.509	**0.453**	0.342	**0.607**	**0.335**	0.606	**1.224**
Correlation													
ARGO-C	**0.874**	0.886	0.923	0.831	0.848	0.797	**0.601**	0.649	0.642	0.731	**0.432**	**0.745**	0.847
ARGOX	0.874	0.886	0.924	**0.832**	**0.851**	0.791	0.592	0.664	**0.688**	0.738	0.403	0.742	0.846
VAR1	0.850	0.867	0.924	0.807	0.819	**0.816**	0.552	0.557	0.486	0.774	0.398	0.700	0.864
GFT	–	–	–	0.766	0.833	–	–	–	–	–	–	–	–
naive	0.873	**0.888**	**0.928**	0.816	0.828	0.810	0.562	**0.721**	0.605	**0.787**	0.390	0.700	**0.884**

Table S10-9. Comparison of different methods for state-level %ILI estimation in District of Columbia. The MSE, MAE, and correlation are reported. The method with the best performance is highlighted in boldface for each metric in each period.

	Whole period	’14-’23	post-COVID	GFT period	’14-’15	’15-’16	’16-’17	’17-’18	’18-’19	’19-’20	’20-’21	’21-’22	’22-’23
RMSE													
ARGO-C	**0.577**	**0.543**	**0.473**	0.860	0.945	**0.318**	**0.421**	1.031	**0.401**	**0.700**	0.247	**0.446**	**0.856**
ARGOX	0.593	0.560	0.492	0.871	0.957	0.330	0.437	**1.017**	0.459	0.753	0.249	0.482	0.951
VAR1	3.205	2.992	2.550	2.220	2.421	1.629	3.280	5.718	3.521	4.928	1.583	2.289	4.099
GFT	–	–	–	**0.819**	**0.896**	–	–	–	–	–	–	–	–
naive	0.816	0.758	0.638	0.999	1.098	0.358	0.564	1.501	0.672	1.360	**0.206**	0.740	0.935
MAE													
ARGO-C	**0.330**	**0.326**	**0.318**	**0.418**	**0.480**	**0.239**	**0.343**	0.622	**0.300**	**0.544**	0.204	0.313	**0.655**
ARGOX	0.339	0.332	0.319	0.430	0.497	0.243	0.368	**0.600**	0.314	0.574	0.201	**0.292**	0.739
VAR1	2.431	2.339	2.170	1.560	1.741	1.554	3.056	4.344	3.335	4.445	1.539	1.950	3.503
GFT	–	–	–	0.505	0.574	–	–	–	–	–	–	–	–
naive	0.430	0.419	0.397	0.486	0.564	0.275	0.418	0.856	0.488	1.041	**0.164**	0.461	0.700
Correlation													
ARGO-C	**0.970**	**0.968**	**0.961**	0.864	0.845	**0.870**	**0.950**	0.969	**0.961**	**0.965**	0.841	**0.971**	**0.897**
ARGOX	0.969	0.966	0.958	0.854	0.833	0.866	0.947	**0.970**	0.950	0.960	0.828	0.960	0.874
VAR1	0.543	0.551	0.662	0.185	0.093	0.622	0.665	0.526	0.647	0.587	0.423	0.770	-0.188
GFT	–	–	–	**0.892**	**0.877**	–	–	–	–	–	–	–	–
naive	0.941	0.939	0.929	0.812	0.787	0.837	0.909	0.933	0.878	0.858	**0.872**	0.885	0.869

Table S10-10. Comparison of different methods for state-level %ILI estimation in Georgia. The MSE, MAE, and correlation are reported. The method with the best performance is highlighted in boldface for each metric in each period.

	Whole period	’14-’23	post-COVID	GFT period	’14-’15	’15-’16	’16-’17	’17-’18	’18-’19	’19-’20	’20-’21	’21-’22	’22-’23
RMSE													
ARGO-C	**0.903**	**0.775**	0.450	1.659	1.702	**0.769**	0.688	**1.155**	**0.611**	0.648	0.354	0.349	**0.471**
ARGOX	0.906	0.778	0.453	1.660	1.704	0.773	**0.684**	1.164	0.625	0.643	**0.350**	0.356	0.485
VAR1	2.945	2.647	1.978	5.999	6.525	1.870	0.973	3.918	1.713	3.541	1.481	1.985	2.306
GFT	–	–	–	4.131	4.457	–	–	–	–	–	–	–	–
naive	0.930	0.796	**0.449**	**1.597**	**1.627**	0.787	0.740	1.269	0.707	**0.618**	0.374	**0.347**	0.504
MAE													
ARGO-C	**0.618**	**0.517**	0.330	1.132	1.185	**0.611**	0.568	0.865	**0.468**	0.487	0.297	0.241	**0.369**
ARGOX	0.620	0.519	0.332	1.137	1.192	0.621	**0.564**	**0.861**	0.474	0.492	**0.295**	0.246	0.379
VAR1	1.636	1.632	1.623	4.806	5.521	1.371	0.747	2.152	1.262	2.811	1.397	1.668	1.804
GFT	–	–	–	3.590	3.997	–	–	–	–	–	–	–	–
naive	0.654	0.540	**0.328**	**1.100**	**1.153**	0.642	0.609	0.977	0.510	**0.475**	0.311	**0.236**	0.398
Correlation													
ARGO-C	**0.935**	**0.942**	0.925	0.915	0.900	**0.907**	**0.745**	**0.770**	**0.870**	**0.905**	0.108	0.862	**0.558**
ARGOX	0.934	0.942	0.924	0.915	0.901	0.905	0.743	0.762	0.864	0.901	0.111	0.860	0.532
VAR1	0.299	0.310	0.700	0.490	0.399	0.796	0.708	0.528	0.457	0.491	-0.050	0.614	0.445
GFT	–	–	–	0.906	**0.942**	–	–	–	–	–	–	–	–
naive	0.932	0.940	**0.927**	**0.923**	0.908	0.905	0.716	0.721	0.833	0.890	**0.113**	**0.865**	0.470

Table S10-11. Comparison of different methods for state-level %ILI estimation in Hawaii. The MSE, MAE, and correlation are reported. The method with the best performance is highlighted in boldface for each metric in each period.

	Whole period	’14-’23	post-COVID	GFT period	’14-’15	’15-’16	’16-’17	’17-’18	’18-’19	’19-’20	’20-’21	’21-’22	’22-’23
RMSE													
ARGO-C	0.508	**0.622**	**0.791**	0.651	0.670	0.530	**0.411**	0.568	**0.730**	0.906	0.233	0.578	**1.475**
ARGOX	**0.495**	0.623	0.809	**0.632**	**0.651**	0.499	0.420	**0.519**	0.739	0.938	**0.231**	**0.569**	1.515
VAR1	2.281	2.027	1.443	4.065	4.298	2.339	1.798	2.449	1.559	1.851	0.500	1.146	3.160
GFT	–	–	–	1.243	1.355	–	–	–	–	–	–	–	–
naive	0.566	0.669	0.826	0.827	0.850	**0.470**	0.478	0.639	0.820	**0.808**	0.234	0.595	1.672
MAE													
ARGO-C	0.361	0.394	0.456	0.475	0.477	0.435	0.330	0.429	**0.522**	0.622	0.153	0.405	**0.976**
ARGOX	**0.347**	**0.389**	0.465	**0.451**	**0.452**	0.401	**0.329**	**0.388**	0.527	0.643	**0.146**	0.398	1.018
VAR1	1.860	1.545	0.962	3.381	3.654	1.923	1.676	2.294	1.379	1.624	0.403	0.938	2.588
GFT	–	–	–	1.035	1.168	–	–	–	–	–	–	–	–
naive	0.380	0.402	**0.442**	0.623	0.632	**0.375**	0.362	0.474	0.583	**0.528**	0.162	**0.376**	1.128
Correlation													
ARGO-C	0.916	**0.900**	**0.871**	0.939	0.931	0.563	**0.801**	0.846	**0.786**	0.685	0.808	0.645	**0.902**
ARGOX	**0.920**	0.899	0.864	0.941	0.933	0.611	0.786	**0.870**	0.778	0.669	0.814	0.653	0.891
VAR1	0.794	0.508	0.528	0.719	0.652	0.587	0.637	0.791	0.679	-0.270	0.428	0.283	0.430
GFT	–	–	–	**0.953**	**0.955**	–	–	–	–	–	–	–	–
naive	0.899	0.890	0.868	0.901	0.888	**0.692**	0.750	0.814	0.754	**0.781**	**0.822**	**0.674**	0.867

Table S10-12. Comparison of different methods for state-level %ILI estimation in Idaho. The MSE, MAE, and correlation are reported. The method with the best performance is highlighted in boldface for each metric in each period.

	Whole period	'14-'23	post-COVID	GFT period	'14-'15	'15-'16	'16-'17	'17-'18	'18-'19	'19-'20	'20-'21	'21-'22	'22-'23
RMSE													
ARGO-C	**0.284**	**0.284**	**0.284**	0.460	0.503	0.233	**0.230**	**0.328**	**0.269**	**0.430**	0.156	0.293	**0.555**
ARGOX	0.291	0.292	0.292	**0.443**	**0.485**	**0.233**	0.241	0.354	0.272	0.471	0.146	**0.273**	0.615
VAR1	1.628	1.552	1.399	2.173	2.332	1.926	1.832	1.962	0.955	2.258	0.715	0.730	2.283
GFT	–	–	–	0.672	0.704	–	–	–	–	–	–	–	–
naive	0.401	0.396	0.387	0.459	0.501	0.271	0.360	0.626	0.393	0.765	**0.114**	0.319	0.756
MAE													
ARGO-C	**0.182**	**0.182**	**0.180**	0.280	0.314	0.189	**0.186**	**0.218**	**0.184**	**0.308**	0.127	0.217	**0.413**
ARGOX	0.188	0.186	0.182	**0.275**	**0.311**	**0.183**	0.197	0.231	0.200	0.338	0.122	**0.212**	0.459
VAR1	1.330	1.175	0.888	1.792	1.926	1.814	1.755	1.609	0.829	1.959	0.382	0.576	2.074
GFT	–	–	–	0.604	0.633	–	–	–	–	–	–	–	–
naive	0.251	0.241	0.223	0.287	0.318	0.221	0.268	0.406	0.285	0.579	**0.091**	0.223	0.613
Correlation													
ARGO-C	**0.979**	**0.979**	**0.978**	0.938	0.925	0.954	**0.976**	**0.983**	**0.953**	**0.977**	0.910	0.922	**0.943**
ARGOX	0.978	0.978	0.977	0.939	0.924	**0.956**	0.972	0.980	0.950	0.972	**0.920**	**0.928**	0.929
VAR1	0.750	0.541	0.501	0.195	-0.003	0.588	0.913	0.860	0.818	0.844	0.726	0.485	0.349
GFT	–	–	–	**0.945**	**0.937**	–	–	–	–	–	–	–	–
naive	0.959	0.960	0.962	0.931	0.914	0.939	0.936	0.937	0.889	0.922	0.920	0.895	0.892

Table S10-13. Comparison of different methods for state-level %ILI estimation in Illinois. The MSE, MAE, and correlation are reported. The method with the best performance is highlighted in boldface for each metric in each period.

	Whole period	'14-'23	post-COVID	GFT period	'14-'15	'15-'16	'16-'17	'17-'18	'18-'19	'19-'20	'20-'21	'21-'22	'22-'23
RMSE													
ARGO-C	**0.544**	**0.512**	**0.446**	0.656	0.714	**0.416**	0.718	**0.571**	**0.596**	**0.810**	0.163	0.230	**0.690**
ARGOX	0.566	0.537	0.478	0.714	0.778	0.431	**0.713**	0.640	0.613	0.884	0.165	**0.207**	0.728
VAR1	1.770	1.584	1.162	1.788	1.936	0.968	1.866	2.985	1.888	2.675	0.172	0.633	2.147
GFT	–	–	–	**0.478**	**0.521**	–	–	–	–	–	–	–	–
naive	0.728	0.654	0.486	0.916	1.004	0.562	0.830	0.978	0.783	1.018	**0.103**	0.248	0.863
MAE													
ARGO-C	**0.370**	**0.333**	**0.264**	0.422	0.464	**0.299**	0.508	**0.412**	0.430	0.540	0.126	0.157	**0.523**
ARGOX	0.377	0.339	0.268	0.465	0.518	0.303	**0.499**	0.459	**0.428**	**0.516**	0.133	**0.144**	0.564
VAR1	1.240	1.057	0.717	1.341	1.494	0.727	1.294	2.375	1.547	2.281	0.131	0.481	1.727
GFT	–	–	–	**0.379**	**0.429**	–	–	–	–	–	–	–	–
naive	0.457	0.397	0.285	0.525	0.607	0.433	0.596	0.624	0.540	0.664	**0.080**	0.193	0.675
Correlation													
ARGO-C	**0.951**	**0.948**	**0.936**	0.937	0.931	**0.870**	0.896	**0.974**	**0.915**	**0.927**	0.911	**0.963**	**0.939**
ARGOX	0.947	0.943	0.928	0.923	0.915	0.860	**0.898**	0.967	0.909	0.913	0.922	0.957	0.929
VAR1	0.515	0.505	0.663	0.511	0.444	0.447	0.604	0.878	0.667	-0.235	0.878	0.189	0.771
GFT	–	–	–	**0.970**	**0.968**	–	–	–	–	–	–	–	–
naive	0.914	0.917	0.926	0.877	0.864	0.776	0.865	0.923	0.857	0.873	**0.934**	0.913	0.901

Table S10-14. Comparison of different methods for state-level %ILI estimation in Indiana. The MSE, MAE, and correlation are reported. The method with the best performance is highlighted in boldface for each metric in each period.

	Whole period	'14-'23	post-COVID	GFT period	'14-'15	'15-'16	'16-'17	'17-'18	'18-'19	'19-'20	'20-'21	'21-'22	'22-'23
RMSE													
ARGO-C	0.399	**0.686**	**1.023**	0.668	0.735	0.280	**0.362**	0.394	0.409	**0.537**	0.930	**0.534**	**1.034**
ARGOX	**0.397**	0.699	1.050	0.652	0.718	**0.267**	0.388	**0.386**	**0.408**	0.632	0.923	0.597	1.119
VAR1	1.442	1.466	1.510	1.982	2.101	1.811	1.730	1.630	0.869	2.133	**0.915**	0.985	1.368
GFT	–	–	–	2.296	2.519	–	–	–	–	–	–	–	–
naive	0.463	0.786	1.169	**0.646**	**0.711**	0.294	0.425	0.554	0.478	0.749	0.995	0.608	1.386
MAE													
ARGO-C	0.244	**0.319**	**0.457**	0.355	0.420	0.236	**0.260**	0.304	**0.288**	**0.362**	0.537	**0.383**	**0.756**
ARGOX	**0.240**	0.324	0.479	0.347	0.414	**0.223**	0.273	**0.285**	0.298	0.396	0.546	0.426	0.802
VAR1	1.206	1.109	0.928	1.643	1.721	1.740	1.577	1.348	0.755	1.834	0.463	0.795	1.110
GFT	–	–	–	1.346	1.534	–	–	–	–	–	–	–	–
naive	0.261	0.350	0.514	**0.314**	**0.377**	0.244	0.302	0.366	0.347	0.466	**0.419**	0.429	1.041
Correlation													
ARGO-C	0.917	**0.845**	**0.759**	0.677	0.600	0.706	**0.907**	0.939	**0.925**	**0.926**	0.495	**0.833**	**0.880**
ARGOX	**0.918**	0.838	0.745	0.683	0.606	0.731	0.892	**0.940**	0.924	0.902	0.508	0.791	0.854
VAR1	0.790	0.592	0.502	0.396	0.261	**0.802**	0.710	0.886	0.821	0.781	0.488	0.379	0.835
GFT	–	–	–	0.575	0.507	–	–	–	–	–	–	–	–
naive	0.891	0.811	0.725	**0.723**	**0.668**	0.702	0.875	0.878	0.895	0.852	**0.516**	0.796	0.784

Table S10-15. Comparison of different methods for state-level %ILI estimation in Iowa. The MSE, MAE, and correlation are reported. The method with the best performance is highlighted in boldface for each metric in each period.

	Whole period	'14-'23	post-COVID	GFT period	'14-'15	'15-'16	'16-'17	'17-'18	'18-'19	'19-'20	'20-'21	'21-'22	'22-'23
RMSE													
ARGO-C	**0.523**	**0.508**	**0.478**	**0.702**	**0.770**	0.283	**0.864**	0.533	0.507	**0.635**	0.212	0.482	**0.931**
ARGOX	0.526	0.515	0.494	0.729	0.799	**0.277**	0.902	**0.481**	**0.456**	0.668	0.213	0.472	1.012
VAR1	1.444	1.251	0.774	1.783	1.958	0.470	2.102	2.230	0.877	1.951	0.249	0.614	1.280
GFT	–	–	–	1.472	1.620	–	–	–	–	–	–	–	–
naive	0.678	0.633	0.542	0.898	0.986	0.298	0.971	1.021	0.554	0.874	**0.196**	**0.441**	1.151
MAE													
ARGO-C	0.332	0.322	**0.303**	0.420	0.487	0.230	**0.569**	0.402	0.320	**0.503**	0.157	0.352	**0.623**
ARGOX	**0.328**	**0.320**	0.305	**0.412**	**0.475**	0.229	0.590	**0.361**	**0.307**	0.516	0.157	0.349	0.676
VAR1	0.879	0.751	0.513	1.168	1.366	0.350	1.201	1.638	0.643	1.542	0.220	0.459	0.925
GFT	–	–	–	1.090	1.302	–	–	–	–	–	–	–	–
naive	0.404	0.376	0.326	0.528	0.614	**0.222**	0.674	0.723	0.381	0.649	**0.142**	**0.323**	0.860
Correlation													
ARGO-C	**0.979**	**0.974**	**0.942**	**0.964**	0.956	0.884	**0.944**	0.990	0.949	**0.970**	0.892	**0.788**	**0.903**
ARGOX	0.979	0.973	0.939	0.962	0.953	**0.890**	0.939	**0.992**	**0.960**	0.968	0.891	0.778	0.882
VAR1	0.883	0.863	0.845	0.860	0.845	0.764	0.758	0.957	0.836	0.909	**0.923**	0.688	0.814
GFT	–	–	–	0.960	**0.957**	–	–	–	–	–	–	–	–
naive	0.964	0.960	0.931	0.939	0.924	0.874	0.929	0.964	0.937	0.943	0.899	0.785	0.850

Table S10-16. Comparison of different methods for state-level %ILI estimation in Kansas. The MSE, MAE, and correlation are reported. The method with the best performance is highlighted in boldface for each metric in each period.

	Whole period	'14-'23	post-COVID	GFT period	'14-'15	'15-'16	'16-'17	'17-'18	'18-'19	'19-'20	'20-'21	'21-'22	'22-'23
RMSE													
ARGO-C	**0.670**	**0.631**	**0.550**	**0.279**	**0.306**	0.254	**1.071**	1.018	**0.837**	**0.997**	0.215	0.391	**1.210**
ARGOX	0.683	0.656	0.603	0.293	0.322	**0.244**	1.075	**0.996**	0.875	1.163	0.217	**0.347**	1.280
VAR1	1.698	1.507	1.067	2.646	2.906	0.741	1.284	1.900	2.522	2.259	0.275	0.636	2.434
GFT	–	–	–	2.912	3.180	–	–	–	–	–	–	–	–
naive	0.850	0.774	0.607	0.393	0.432	0.281	1.222	1.300	1.038	1.384	**0.174**	0.395	1.438
MAE													
ARGO-C	**0.379**	**0.353**	0.306	**0.178**	**0.206**	0.164	**0.798**	0.692	**0.504**	**0.658**	0.168	0.306	**0.860**
ARGOX	0.391	0.361	**0.306**	0.188	0.216	**0.158**	0.830	**0.668**	0.533	0.746	0.171	**0.261**	0.907
VAR1	1.019	0.869	0.590	2.027	2.365	0.697	0.834	1.229	1.544	1.768	0.235	0.464	1.738
GFT	–	–	–	2.101	2.363	–	–	–	–	–	–	–	–
naive	0.465	0.420	0.337	0.220	0.265	0.190	0.913	0.871	0.680	0.975	**0.136**	0.324	1.099
Correlation													
ARGO-C	**0.971**	**0.968**	**0.953**	0.952	0.947	0.925	**0.925**	0.965	**0.957**	**0.955**	0.848	0.921	**0.903**
ARGOX	0.970	0.965	0.945	0.946	0.941	**0.931**	0.924	**0.968**	0.953	0.940	0.860	**0.923**	0.889
VAR1	0.797	0.797	0.800	0.777	0.749	0.916	0.894	0.872	0.723	0.772	0.808	0.728	0.657
GFT	–	–	–	**0.962**	**0.959**	–	–	–	–	–	–	–	–
naive	0.954	0.952	0.943	0.906	0.895	0.908	0.898	0.941	0.934	0.909	**0.862**	0.859	0.867

Table S10-17. Comparison of different methods for state-level %ILI estimation in Kentucky. The MSE, MAE, and correlation are reported. The method with the best performance is highlighted in boldface for each metric in each period.

	Whole period	'14-'23	post-COVID	GFT period	'14-'15	'15-'16	'16-'17	'17-'18	'18-'19	'19-'20	'20-'21	'21-'22	'22-'23
RMSE													
ARGO-C	**0.511**	**0.487**	0.438	0.488	0.534	0.276	**0.274**	**0.617**	0.870	**0.896**	0.507	0.350	**0.569**
ARGOX	0.524	0.496	**0.438**	**0.455**	**0.498**	0.264	0.277	0.679	**0.866**	0.937	0.491	**0.317**	0.655
VAR1	2.297	2.099	1.671	2.946	3.199	1.567	2.045	2.380	1.876	4.363	0.345	0.769	2.875
GFT	–	–	–	1.015	1.106	–	–	–	–	–	–	–	–
naive	0.641	0.612	0.553	0.567	0.621	**0.249**	0.355	0.917	0.981	1.373	**0.323**	0.385	0.680
MAE													
ARGO-C	**0.306**	**0.299**	**0.287**	0.325	0.371	0.230	**0.200**	**0.429**	**0.491**	**0.682**	0.318	0.225	**0.471**
ARGOX	0.318	0.308	0.290	**0.310**	**0.355**	0.220	0.215	0.476	0.515	0.741	0.303	**0.198**	0.562
VAR1	1.888	1.599	1.065	2.661	2.979	1.501	1.598	2.032	1.666	3.708	0.289	0.686	2.146
GFT	–	–	–	0.748	0.838	–	–	–	–	–	–	–	–
naive	0.386	0.361	0.315	0.384	0.441	**0.195**	0.272	0.608	0.705	1.121	**0.144**	0.267	0.592
Correlation													
ARGO-C	**0.979**	**0.980**	0.976	0.964	0.952	0.871	**0.968**	**0.980**	**0.951**	**0.952**	0.252	**0.955**	**0.898**
ARGOX	0.978	0.979	**0.976**	0.968	0.955	**0.878**	0.968	0.976	0.950	0.948	0.244	0.951	0.866
VAR1	0.769	0.711	0.621	0.735	0.708	0.725	0.714	0.774	0.906	0.659	**0.442**	0.775	0.408
GFT	–	–	–	**0.981**	**0.978**	–	–	–	–	–	–	–	–
naive	0.968	0.968	0.965	0.946	0.924	0.876	0.948	0.952	0.927	0.884	0.422	0.878	0.849

Table S10-18. Comparison of different methods for state-level %ILI estimation in Louisiana. The MSE, MAE, and correlation are reported. The method with the best performance is highlighted in boldface for each metric in each period.

	Whole period	'14-'23	post-COVID	GFT period	'14-'15	'15-'16	'16-'17	'17-'18	'18-'19	'19-'20	'20-'21	'21-'22	'22-'23
RMSE													
ARGO-C	**0.276**	**0.391**	**0.542**	**0.327**	**0.352**	0.335	**0.274**	**0.248**	**0.293**	**0.828**	0.127	0.287	**1.136**
ARGOX	0.278	0.398	0.556	0.328	0.353	**0.332**	0.280	0.251	0.296	0.857	0.117	0.317	1.142
VAR1	3.012	2.731	2.112	2.174	2.376	1.319	2.017	5.069	3.685	5.899	0.617	0.840	2.727
GFT	–	–	–	0.744	0.813	–	–	–	–	–	–	–	–
naive	0.296	0.428	0.602	0.354	0.380	0.375	0.297	0.253	0.297	0.865	**0.114**	**0.255**	1.354
MAE													
ARGO-C	**0.203**	**0.222**	**0.257**	0.253	0.273	0.269	**0.216**	**0.197**	0.236	**0.440**	0.099	0.218	**0.805**
ARGOX	0.204	0.224	0.262	**0.252**	**0.272**	**0.266**	0.220	0.197	0.238	0.453	0.093	0.230	0.811
VAR1	2.244	1.931	1.350	1.792	2.047	1.229	1.786	4.150	3.133	5.433	0.514	0.680	2.165
GFT	–	–	–	0.482	0.547	–	–	–	–	–	–	–	–
naive	0.216	0.238	0.278	0.273	0.292	0.290	0.229	0.208	**0.232**	0.451	**0.091**	**0.193**	1.034
Correlation													
ARGO-C	**0.952**	**0.944**	**0.940**	0.886	0.859	0.295	**0.804**	**0.792**	**0.945**	**0.894**	0.693	0.878	0.921
ARGOX	0.951	0.943	0.939	0.885	0.858	0.306	0.793	0.787	0.943	0.887	**0.702**	0.856	**0.921**
VAR1	0.677	0.534	0.506	0.696	0.600	**0.328**	0.497	0.625	0.845	0.571	0.281	0.211	0.272
GFT	–	–	–	**0.917**	**0.916**	–	–	–	–	–	–	–	–
naive	0.945	0.933	0.926	0.871	0.844	0.257	0.789	0.760	0.940	0.885	0.645	**0.897**	0.882

Table S10-19. Comparison of different methods for state-level %ILI estimation in Maine. The MSE, MAE, and correlation are reported. The method with the best performance is highlighted in boldface for each metric in each period.

	Whole period	'14-'23	post-COVID	GFT period	'14-'15	'15-'16	'16-'17	'17-'18	'18-'19	'19-'20	'20-'21	'21-'22	'22-'23
RMSE													
ARGO-C	**0.479**	**0.462**	**0.431**	**0.540**	**0.514**	**0.568**	**0.593**	0.446	0.511	0.646	0.135	**0.428**	**0.791**
ARGOX	0.494	0.476	0.440	0.558	0.522	0.580	0.621	**0.438**	**0.510**	**0.639**	**0.126**	0.459	0.893
VAR1	1.055	1.168	1.352	1.089	1.098	1.237	1.329	0.993	0.775	1.705	0.417	0.806	3.443
GFT	–	–	–	1.282	1.387	–	–	–	–	–	–	–	–
naive	0.601	0.565	0.491	0.714	0.743	0.657	0.721	0.619	0.624	0.810	0.134	0.564	0.893
MAE													
ARGO-C	**0.353**	**0.318**	**0.253**	0.364	0.326	**0.440**	**0.463**	**0.336**	0.403	**0.451**	0.108	**0.278**	**0.638**
ARGOX	0.362	0.327	0.262	**0.359**	**0.311**	0.450	0.492	0.348	**0.402**	0.498	**0.103**	0.280	0.745
VAR1	0.768	0.791	0.832	0.730	0.674	0.990	0.939	0.672	0.640	1.279	0.377	0.642	3.007
GFT	–	–	–	1.023	1.137	–	–	–	–	–	–	–	–
naive	0.416	0.371	0.289	0.447	0.434	0.494	0.510	0.444	0.496	0.620	0.106	0.335	0.743
Correlation													
ARGO-C	**0.942**	**0.950**	**0.962**	**0.859**	0.894	**0.745**	**0.924**	0.963	0.914	0.956	0.916	**0.924**	**0.900**
ARGOX	0.939	0.947	0.960	0.841	0.884	0.730	0.913	**0.964**	**0.915**	**0.957**	**0.920**	0.905	0.875
VAR1	0.765	0.714	0.687	0.358	0.396	0.223	0.849	0.794	0.814	0.789	0.824	0.694	-0.081
GFT	–	–	–	0.853	**0.926**	–	–	–	–	–	–	–	–
naive	0.911	0.928	0.952	0.753	0.775	0.695	0.872	0.924	0.876	0.918	0.905	0.838	0.869

Table S10-20. Comparison of different methods for state-level %ILI estimation in Maryland. The MSE, MAE, and correlation are reported. The method with the best performance is highlighted in boldface for each metric in each period.

	Whole period	'14-'23	post-COVID	GFT period	'14-'15	'15-'16	'16-'17	'17-'18	'18-'19	'19-'20	'20-'21	'21-'22	'22-'23
RMSE													
ARGO-C	**0.242**	**0.326**	**0.442**	0.275	**0.296**	0.230	**0.190**	**0.369**	**0.188**	0.621	0.115	**0.426**	**0.897**
ARGOX	0.249	0.331	0.446	**0.275**	0.297	**0.223**	0.204	0.381	0.213	**0.619**	0.101	0.445	0.911
VAR1	0.720	0.864	1.080	0.648	0.621	0.816	0.892	0.419	0.652	1.012	0.415	0.920	2.514
GFT	–	–	–	0.344	0.353	–	–	–	–	–	–	–	–
naive	0.329	0.415	0.540	0.335	0.363	0.258	0.271	0.541	0.324	0.711	**0.085**	0.500	1.229
MAE													
ARGO-C	**0.161**	**0.188**	**0.239**	0.186	0.196	0.171	**0.154**	**0.263**	**0.146**	**0.393**	0.092	**0.311**	**0.604**
ARGOX	0.163	0.191	0.242	**0.177**	**0.189**	**0.163**	0.159	0.276	0.162	0.404	0.079	0.326	0.635
VAR1	0.577	0.638	0.752	0.506	0.461	0.681	0.653	0.316	0.540	0.835	0.386	0.756	1.997
GFT	–	–	–	0.266	0.260	–	–	–	–	–	–	–	–
naive	0.208	0.234	0.282	0.237	0.258	0.181	0.203	0.368	0.231	0.550	**0.061**	0.376	0.861
Correlation													
ARGO-C	**0.975**	**0.970**	**0.966**	0.916	0.908	0.888	**0.954**	**0.965**	**0.973**	0.936	0.918	**0.922**	**0.952**
ARGOX	0.973	0.969	0.965	0.920	0.913	**0.894**	0.947	0.963	0.964	0.938	0.923	0.913	0.948
VAR1	0.846	0.824	0.806	0.534	0.571	0.533	0.819	0.950	0.801	**0.943**	0.879	0.590	0.426
GFT	–	–	–	**0.940**	**0.931**	–	–	–	–	–	–	–	–
naive	0.952	0.949	0.946	0.877	0.861	0.870	0.908	0.919	0.919	0.912	**0.927**	0.890	0.892

Table S10-21. Comparison of different methods for state-level %ILI estimation in Massachusetts. The MSE, MAE, and correlation are reported. The method with the best performance is highlighted in boldface for each metric in each period.

	Whole period	'14-'23	post-COVID	GFT period	'14-'15	'15-'16	'16-'17	'17-'18	'18-'19	'19-'20	'20-'21	'21-'22	'22-'23
RMSE													
ARGO-C	0.276	**0.250**	**0.194**	**0.366**	**0.398**	0.207	0.332	0.338	0.298	**0.316**	0.132	**0.223**	0.329
ARGOX	**0.275**	0.252	0.201	0.384	0.419	**0.199**	**0.328**	**0.319**	**0.285**	0.344	**0.130**	0.238	**0.326**
VAR1	1.020	1.029	1.046	0.980	1.048	0.646	1.471	2.000	0.605	1.188	0.173	0.408	2.644
GFT	–	–	–	1.203	1.302	–	–	–	–	–	–	–	–
naive	0.359	0.329	0.266	0.538	0.591	0.257	0.411	0.489	0.329	0.437	0.132	0.245	0.520
MAE													
ARGO-C	**0.193**	**0.173**	**0.137**	**0.247**	**0.278**	0.165	**0.226**	0.273	0.226	**0.230**	0.106	**0.163**	0.255
ARGOX	0.194	0.175	0.140	0.264	0.302	**0.158**	0.238	**0.248**	**0.221**	0.259	0.108	0.171	**0.253**
VAR1	0.673	0.610	0.493	0.741	0.783	0.447	1.138	1.582	0.501	0.760	0.146	0.331	1.845
GFT	–	–	–	1.017	1.126	–	–	–	–	–	–	–	–
naive	0.234	0.212	0.171	0.304	0.353	0.184	0.299	0.403	0.255	0.321	**0.103**	0.176	0.412
Correlation													
ARGO-C	0.967	**0.969**	**0.972**	0.939	0.931	0.950	0.947	0.973	0.861	**0.962**	0.238	**0.855**	0.939
ARGOX	**0.967**	0.969	0.971	0.932	0.925	**0.957**	**0.949**	**0.977**	**0.875**	0.954	0.254	0.835	**0.940**
VAR1	0.479	0.542	0.848	0.382	0.250	0.124	0.333	0.627	0.632	0.812	**0.389**	0.620	0.816
GFT	–	–	–	**0.978**	**0.976**	–	–	–	–	–	–	–	–
naive	0.944	0.947	0.949	0.860	0.839	0.917	0.917	0.936	0.837	0.924	0.174	0.804	0.849

Table S10-22. Comparison of different methods for state-level %ILI estimation in Michigan. The MSE, MAE, and correlation are reported. The method with the best performance is highlighted in boldface for each metric in each period.

	Whole period	'14-'23	post-COVID	GFT period	'14-'15	'15-'16	'16-'17	'17-'18	'18-'19	'19-'20	'20-'21	'21-'22	'22-'23
RMSE													
ARGO-C	**0.571**	**0.568**	**0.561**	**0.611**	**0.658**	**0.560**	**0.415**	0.407	**0.503**	**1.187**	0.239	**0.963**	**0.666**
ARGOX	0.578	0.593	0.619	0.653	0.706	0.565	0.420	**0.375**	0.505	1.248	0.253	1.016	0.768
VAR1	1.153	1.164	1.185	1.557	1.693	0.758	0.812	0.934	1.185	2.129	0.198	2.103	1.641
GFT	–	–	–	0.780	0.850	–	–	–	–	–	–	–	–
naive	0.721	0.671	0.567	0.898	0.970	0.714	0.526	0.502	0.538	1.437	**0.092**	0.984	0.770
MAE													
ARGO-C	**0.362**	**0.342**	0.306	**0.417**	**0.454**	0.476	**0.318**	0.330	**0.363**	**0.654**	0.182	0.575	**0.438**
ARGOX	0.365	0.356	0.339	0.455	0.498	**0.475**	0.331	**0.310**	0.364	0.678	0.202	0.626	0.548
VAR1	0.816	0.764	0.666	1.156	1.283	0.565	0.681	0.757	1.002	1.679	0.149	1.509	1.275
GFT	–	–	–	0.573	0.653	–	–	–	–	–	–	–	–
naive	0.431	0.375	**0.271**	0.584	0.641	0.544	0.378	0.392	0.402	0.775	**0.076**	**0.475**	0.563
Correlation													
ARGO-C	**0.924**	**0.926**	0.924	**0.928**	**0.919**	**0.741**	**0.936**	0.976	**0.835**	**0.786**	0.665	0.827	**0.901**
ARGOX	0.922	0.919	0.908	0.917	0.905	0.734	0.936	**0.979**	0.835	0.760	0.688	0.806	0.869
VAR1	0.814	0.786	0.704	0.791	0.755	0.689	0.768	0.957	0.500	0.684	0.055	0.532	0.241
GFT	–	–	–	0.927	0.916	–	–	–	–	–	–	–	–
naive	0.882	0.900	**0.926**	0.844	0.825	0.629	0.888	0.959	0.807	0.670	**0.864**	**0.829**	0.861

Table S10-23. Comparison of different methods for state-level %ILI estimation in Minnesota. The MSE, MAE, and correlation are reported. The method with the best performance is highlighted in boldface for each metric in each period.

	Whole period	'14-'23	post-COVID	GFT period	'14-'15	'15-'16	'16-'17	'17-'18	'18-'19	'19-'20	'20-'21	'21-'22	'22-'23
RMSE													
ARGO-C	**0.568**	**0.561**	**0.547**	**0.652**	**0.713**	**0.353**	**0.486**	**0.939**	**0.632**	**0.691**	**0.400**	**0.428**	**1.131**
ARGOX	0.610	0.604	0.591	0.755	0.828	0.354	0.488	1.027	0.636	0.763	0.400	0.438	1.270
VAR1	2.273	2.162	1.938	2.985	3.274	1.781	1.877	4.023	2.191	2.034	1.610	1.553	3.783
GFT	–	–	–	1.454	1.561	–	–	–	–	–	–	–	–
naive	0.790	0.751	0.673	1.022	1.123	0.448	0.650	1.320	0.840	0.946	0.446	0.634	1.384
MAE													
ARGO-C	**0.381**	**0.387**	**0.398**	**0.467**	**0.531**	**0.285**	**0.388**	**0.666**	**0.411**	**0.555**	**0.313**	0.333	**0.983**
ARGOX	0.403	0.406	0.412	0.519	0.598	0.289	0.393	0.730	0.429	0.595	0.314	**0.316**	1.115
VAR1	1.605	1.600	1.591	2.362	2.740	1.676	1.593	2.812	1.533	1.643	1.499	1.343	3.178
GFT	–	–	–	1.284	1.394	–	–	–	–	–	–	–	–
naive	0.507	0.495	0.473	0.665	0.781	0.344	0.519	0.906	0.601	0.769	0.354	0.442	1.178
Correlation													
ARGO-C	**0.969**	**0.964**	**0.944**	**0.964**	**0.952**	0.891	0.933	**0.966**	**0.958**	**0.939**	0.663	**0.932**	**0.854**
ARGOX	0.964	0.958	0.935	0.951	0.935	**0.891**	**0.933**	0.960	0.957	0.926	**0.663**	0.931	0.820
VAR1	0.681	0.666	0.661	0.706	0.650	0.641	0.638	0.432	0.673	0.659	-0.259	0.550	0.365
GFT	–	–	–	0.956	0.949	–	–	–	–	–	–	–	–
naive	0.940	0.936	0.918	0.910	0.881	0.835	0.883	0.928	0.912	0.874	0.617	0.852	0.784

Table S10-24. Comparison of different methods for state-level %ILI estimation in Mississippi. The MSE, MAE, and correlation are reported. The method with the best performance is highlighted in boldface for each metric in each period.

	Whole period	'14-'23	post-COVID	GFT period	'14-'15	'15-'16	'16-'17	'17-'18	'18-'19	'19-'20	'20-'21	'21-'22	'22-'23
RMSE													
ARGO-C	**0.658**	**0.584**	**0.410**	**0.531**	**0.564**	0.370	**0.624**	0.949	**0.807**	**1.223**	0.164	0.372	**0.553**
ARGOX	0.670	0.608	0.472	0.561	0.600	**0.360**	0.676	**0.946**	0.840	1.335	0.154	**0.331**	0.580
VAR1	0.876	0.759	0.470	0.895	0.971	0.394	0.761	1.378	1.012	1.542	**0.107**	0.396	0.741
GFT	–	–	–	0.728	0.786	–	–	–	–	–	–	–	–
naive	0.843	0.744	0.511	0.911	0.989	0.393	0.692	1.368	1.008	1.474	0.112	0.406	0.726
MAE													
ARGO-C	**0.393**	**0.348**	0.264	**0.355**	**0.370**	0.302	**0.410**	0.614	**0.561**	**0.841**	0.130	0.260	**0.407**
ARGOX	0.396	0.352	0.271	0.375	0.396	**0.284**	0.455	**0.610**	0.571	0.891	0.126	**0.241**	0.423
VAR1	0.488	0.405	**0.249**	0.491	0.542	0.311	0.489	0.884	0.719	1.045	**0.084**	0.259	0.553
GFT	–	–	–	0.604	0.670	–	–	–	–	–	–	–	–
naive	0.484	0.407	0.265	0.528	0.586	0.311	0.466	0.907	0.718	1.035	0.085	0.271	0.533
Correlation													
ARGO-C	**0.960**	**0.960**	**0.966**	**0.961**	0.953	0.771	**0.932**	0.970	**0.940**	**0.929**	0.845	0.961	**0.943**
ARGOX	0.959	0.957	0.958	0.957	0.947	**0.784**	0.920	**0.970**	0.929	0.917	0.846	**0.961**	0.934
VAR1	0.930	0.933	0.947	0.886	0.857	0.756	0.912	0.934	0.886	0.894	**0.908**	0.926	0.894
GFT	–	–	–	0.959	**0.964**	–	–	–	–	–	–	–	–
naive	0.934	0.936	0.946	0.886	0.858	0.761	0.919	0.936	0.890	0.900	0.904	0.919	0.896

Table S10-25. Comparison of different methods for state-level %ILI estimation in Missouri. The MSE, MAE, and correlation are reported. The method with the best performance is highlighted in boldface for each metric in each period.

	Whole period	'14-'23	post-COVID	GFT period	'14-'15	'15-'16	'16-'17	'17-'18	'18-'19	'19-'20	'20-'21	'21-'22	'22-'23
RMSE													
ARGO-C	**0.227**	**0.284**	**0.368**	**0.249**	**0.271**	0.233	**0.141**	0.157	0.439	**0.390**	**0.166**	**0.349**	**0.811**
ARGOX	0.229	0.291	0.381	0.254	0.277	**0.233**	0.143	**0.157**	0.440	0.398	0.167	0.362	0.850
VAR1	3.395	2.943	1.829	4.147	4.503	2.903	3.537	6.030	2.562	2.865	1.351	1.787	3.343
GFT	–	–	–	1.100	1.192	–	–	–	–	–	–	–	–
naive	0.241	0.317	0.424	0.296	0.323	0.259	0.154	0.174	**0.429**	0.457	0.166	0.394	0.953
MAE													
ARGO-C	**0.136**	**0.168**	**0.225**	**0.181**	**0.204**	**0.160**	**0.097**	0.117	**0.320**	**0.276**	**0.123**	**0.262**	**0.557**
ARGOX	0.137	0.169	0.229	0.184	0.208	0.161	0.099	**0.117**	0.321	0.283	0.124	0.265	0.586
VAR1	2.805	2.369	1.559	3.685	4.120	2.838	3.325	5.030	2.358	2.514	1.237	1.619	2.868
GFT	–	–	–	0.930	1.029	–	–	–	–	–	–	–	–
naive	0.140	0.179	0.253	0.188	0.213	0.169	0.112	0.123	0.320	0.325	0.125	0.287	0.682
Correlation													
ARGO-C	**0.961**	**0.958**	**0.942**	0.898	0.887	0.421	**0.575**	0.751	0.919	**0.958**	0.816	**0.818**	**0.885**
ARGOX	0.960	0.956	0.939	0.893	0.881	0.412	0.562	**0.753**	0.919	0.956	0.816	0.808	0.875
VAR1	0.497	0.460	0.735	0.703	0.655	**0.689**	0.276	0.357	**0.945**	0.639	0.087	0.659	0.332
GFT	–	–	–	**0.938**	**0.930**	–	–	–	–	–	–	–	–
naive	0.956	0.948	0.925	0.861	0.845	0.341	0.499	0.725	0.916	0.942	**0.823**	0.766	0.852

Table S10-26. Comparison of different methods for state-level %ILI estimation in Montana. The MSE, MAE, and correlation are reported. The method with the best performance is highlighted in boldface for each metric in each period.

	Whole period	'14-'23	post-COVID	GFT period	'14-'15	'15-'16	'16-'17	'17-'18	'18-'19	'19-'20	'20-'21	'21-'22	'22-'23
RMSE													
ARGO-C	**0.605**	**0.600**	**0.591**	**0.698**	**0.766**	0.326	**0.466**	0.823	**0.828**	**0.789**	0.239	**0.362**	**1.489**
ARGOX	0.617	0.617	0.617	0.707	0.777	0.303	0.492	**0.817**	0.894	0.850	0.230	0.389	1.536
VAR1	2.160	1.991	1.632	1.568	1.711	0.491	1.314	3.915	3.141	2.887	0.415	1.269	4.152
GFT	–	–	–	0.866	0.951	–	–	–	–	–	–	–	–
naive	0.704	0.702	0.697	0.835	0.918	**0.286**	0.521	0.913	0.972	1.100	**0.193**	0.479	1.744
MAE													
ARGO-C	0.388	**0.377**	**0.357**	0.401	0.464	0.259	**0.330**	0.606	**0.538**	**0.575**	0.203	**0.285**	**1.091**
ARGOX	**0.388**	0.382	0.371	**0.397**	**0.461**	0.242	0.343	**0.595**	0.583	0.623	0.195	0.295	1.162
VAR1	1.435	1.287	1.013	1.206	1.356	0.403	0.951	3.412	2.361	2.144	0.353	1.121	3.299
GFT	–	–	–	0.530	0.618	–	–	–	–	–	–	–	–
naive	0.433	0.417	0.387	0.479	0.564	**0.211**	0.353	0.693	0.684	0.692	**0.160**	0.338	1.336
Correlation													
ARGO-C	**0.950**	**0.951**	**0.952**	0.840	0.812	0.536	**0.883**	0.927	**0.927**	**0.942**	0.607	**0.878**	**0.892**
ARGOX	0.948	0.948	0.947	0.838	0.810	0.591	0.870	**0.929**	0.913	0.936	0.628	0.850	0.881
VAR1	0.589	0.623	0.751	0.807	0.776	0.380	0.660	0.662	0.256	0.381	0.462	0.434	0.577
GFT	–	–	–	**0.842**	**0.816**	–	–	–	–	–	–	–	–
naive	0.934	0.935	0.934	0.785	0.752	**0.608**	0.859	0.900	0.901	0.872	**0.674**	0.765	0.846

Table S10-27. Comparison of different methods for state-level %ILI estimation in Nebraska. The MSE, MAE, and correlation are reported. The method with the best performance is highlighted in boldface for each metric in each period.

	Whole period	'14-'23	post-COVID	GFT period	'14-'15	'15-'16	'16-'17	'17-'18	'18-'19	'19-'20	'20-'21	'21-'22	'22-'23
RMSE													
ARGO-C	**0.308**	**0.333**	**0.374**	**0.365**	**0.400**	0.332	**0.245**	**0.215**	**0.368**	0.518	0.403	**0.319**	**0.370**
ARGOX	0.310	0.338	0.384	0.371	0.406	**0.332**	0.254	0.221	0.369	0.527	**0.400**	0.321	0.430
VAR1	1.351	1.313	1.240	0.557	0.580	1.095	2.185	2.248	1.059	1.985	0.479	0.887	2.850
GFT	–	–	–	1.658	1.705	–	–	–	–	–	–	–	–
naive	0.330	0.359	0.408	0.447	0.489	0.363	0.245	0.354	0.429	**0.394**	0.418	0.354	0.550
MAE													
ARGO-C	**0.209**	**0.236**	**0.285**	**0.272**	**0.315**	**0.235**	0.187	**0.174**	0.231	0.418	0.303	0.238	**0.294**
ARGOX	0.210	0.237	0.288	0.278	0.321	0.240	0.195	0.175	**0.230**	0.424	**0.298**	**0.230**	0.325
VAR1	0.830	0.820	0.800	0.447	0.454	0.921	1.507	1.457	0.759	1.626	0.389	0.777	2.361
GFT	–	–	–	1.569	1.602	–	–	–	–	–	–	–	–
naive	0.220	0.248	0.300	0.310	0.356	0.251	**0.185**	0.270	0.283	**0.320**	0.316	0.265	0.423
Correlation													
ARGO-C	**0.940**	**0.931**	**0.902**	0.950	0.941	**0.886**	**0.885**	**0.976**	**0.923**	0.809	**0.700**	**0.860**	**0.939**
ARGOX	0.939	0.929	0.897	**0.950**	**0.942**	0.886	0.872	0.975	0.922	0.803	0.700	0.860	0.919
VAR1	0.618	0.549	0.492	0.909	0.891	0.655	0.281	0.618	0.769	0.057	0.679	0.787	0.635
GFT	–	–	–	0.930	0.919	–	–	–	–	–	–	–	–
naive	0.932	0.922	0.888	0.922	0.909	0.859	0.865	0.930	0.898	**0.866**	0.696	0.827	0.862

Table S10-28. Comparison of different methods for state-level %ILI estimation in Nevada. The MSE, MAE, and correlation are reported. The method with the best performance is highlighted in boldface for each metric in each period.

	Whole period	'14-'23	post-COVID	GFT period	'14-'15	'15-'16	'16-'17	'17-'18	'18-'19	'19-'20	'20-'21	'21-'22	'22-'23
RMSE													
ARGO-C	0.407	**0.407**	0.406	0.285	0.312	0.152	0.340	**0.719**	0.605	0.662	**0.230**	0.584	**0.269**
ARGOX	**0.406**	0.411	0.421	0.272	0.298	**0.148**	**0.327**	0.727	**0.595**	0.709	0.236	**0.581**	0.284
VAR1	0.872	1.390	2.027	1.228	1.350	0.375	0.604	1.318	0.810	1.884	1.259	2.589	3.477
GFT	–	–	–	1.106	1.196	–	–	–	–	–	–	–	–
naive	0.436	0.419	**0.385**	**0.241**	**0.264**	0.165	0.346	0.797	0.612	**0.620**	0.251	0.598	0.366
MAE													
ARGO-C	0.243	**0.240**	0.235	0.183	0.211	0.109	0.227	0.476	**0.363**	**0.423**	**0.161**	0.374	**0.215**
ARGOX	**0.243**	0.243	0.243	0.176	0.204	**0.106**	**0.223**	0.479	0.365	0.453	0.164	0.374	0.229
VAR1	0.541	0.888	1.533	0.802	0.946	0.303	0.439	0.731	0.583	1.497	1.171	1.990	2.725
GFT	–	–	–	0.889	0.972	–	–	–	–	–	–	–	–
naive	0.258	0.248	**0.228**	**0.156**	**0.181**	0.116	0.242	**0.476**	0.384	0.452	0.177	**0.373**	0.274
Correlation													
ARGO-C	0.918	**0.895**	0.807	0.840	0.813	0.897	0.663	**0.909**	0.732	0.919	0.222	0.639	**0.914**
ARGOX	**0.919**	0.893	0.793	0.858	0.835	**0.904**	0.688	0.907	0.743	0.906	0.232	0.636	0.906
VAR1	0.660	0.454	0.530	0.888	0.870	0.636	0.669	0.793	0.473	0.600	**0.497**	-0.197	0.839
GFT	–	–	–	**0.909**	**0.897**	–	–	–	–	–	–	–	–
naive	0.907	0.894	**0.823**	0.894	0.879	0.883	**0.692**	0.886	**0.743**	**0.928**	0.192	**0.661**	0.844

Table S10-29. Comparison of different methods for state-level %ILI estimation in New Hampshire. The MSE, MAE, and correlation are reported. The method with the best performance is highlighted in boldface for each metric in each period.

	Whole period	'14-'23	post-COVID	GFT period	'14-'15	'15-'16	'16-'17	'17-'18	'18-'19	'19-'20	'20-'21	'21-'22	'22-'23
RMSE													
ARGO-C	**0.446**	**0.623**	**0.859**	**0.470**	**0.511**	**0.485**	0.620	**0.716**	**0.196**	**1.575**	0.266	**0.810**	**0.773**
ARGOX	0.461	0.638	0.876	0.498	0.541	0.485	0.676	0.718	0.238	1.590	0.252	0.848	0.812
VAR1	1.548	1.842	2.290	1.157	1.221	1.580	2.953	1.932	1.062	4.069	1.344	1.738	2.497
GFT	–	–	–	0.828	0.860	–	–	–	–	–	–	–	–
naive	0.566	0.704	0.905	0.593	0.641	0.503	**0.605**	1.004	0.431	1.625	**0.234**	0.973	0.947
MAE													
ARGO-C	**0.319**	**0.352**	**0.413**	**0.367**	**0.410**	0.381	0.510	0.526	**0.165**	0.892	0.206	**0.466**	**0.590**
ARGOX	0.322	0.356	0.419	0.386	0.435	0.381	0.552	**0.506**	0.195	**0.876**	0.193	0.485	0.636
VAR1	1.145	1.248	1.439	0.987	1.035	1.231	2.780	1.316	0.941	2.654	1.268	1.166	2.084
GFT	–	–	–	0.688	0.699	–	–	–	–	–	–	–	–
naive	0.361	0.402	0.477	0.438	0.483	**0.376**	**0.456**	0.609	0.303	1.113	**0.182**	0.556	0.783
Correlation													
ARGO-C	**0.972**	**0.959**	**0.944**	**0.895**	**0.879**	0.922	**0.927**	0.969	**0.988**	**0.916**	**0.889**	**0.895**	**0.934**
ARGOX	0.970	0.957	0.942	0.884	0.866	**0.923**	0.910	**0.970**	0.982	0.915	0.882	0.883	0.928
VAR1	0.790	0.728	0.648	0.802	0.771	0.580	0.777	0.827	0.898	0.409	0.370	0.525	0.617
GFT	–	–	–	0.888	0.872	–	–	–	–	–	–	–	–
naive	0.955	0.947	0.937	0.837	0.810	0.909	0.917	0.929	0.940	0.901	0.881	0.831	0.892

Table S10-30. Comparison of different methods for state-level %ILI estimation in New Jersey. The MSE, MAE, and correlation are reported. The method with the best performance is highlighted in boldface for each metric in each period.

	Whole period	'14-'23	post-COVID	GFT period	'14-'15	'15-'16	'16-'17	'17-'18	'18-'19	'19-'20	'20-'21	'21-'22	'22-'23
RMSE													
ARGO-C	0.502	**0.573**	**0.685**	**0.430**	**0.466**	**0.449**	0.362	0.731	0.586	**0.856**	**0.248**	0.535	**1.687**
ARGOX	**0.498**	0.574	0.694	0.449	0.488	0.465	**0.347**	**0.689**	**0.567**	0.928	0.249	**0.498**	1.691
VAR1	2.238	2.942	3.927	2.220	2.402	2.092	1.849	2.396	2.605	4.151	1.208	3.901	9.287
GFT	–	–	–	1.456	1.510	–	–	–	–	–	–	–	–
naive	0.647	0.726	0.852	0.665	0.729	0.484	0.409	0.911	0.749	1.136	0.256	0.706	2.174
MAE													
ARGO-C	0.329	0.355	**0.402**	0.304	0.335	**0.345**	0.267	0.441	0.390	**0.637**	**0.195**	0.412	**1.270**
ARGOX	**0.322**	**0.351**	0.405	**0.299**	**0.331**	0.360	**0.244**	**0.415**	**0.381**	0.678	0.197	**0.393**	1.271
VAR1	1.610	2.016	2.769	1.886	2.080	1.831	1.560	1.692	2.070	3.290	1.137	3.667	7.937
GFT	–	–	–	1.285	1.322	–	–	–	–	–	–	–	–
naive	0.389	0.418	0.471	0.392	0.443	0.368	0.310	0.592	0.501	0.759	0.201	0.533	1.618
Correlation													
ARGO-C	0.967	**0.972**	**0.975**	**0.948**	0.938	**0.929**	0.949	0.952	0.955	**0.962**	**0.837**	0.935	**0.952**
ARGOX	**0.967**	0.971	0.973	0.943	0.933	0.923	**0.953**	**0.958**	**0.957**	0.956	0.834	**0.946**	0.947
VAR1	0.675	0.500	0.493	0.480	0.376	0.661	0.530	0.710	0.696	0.636	0.272	0.455	0.451
GFT	–	–	–	0.947	**0.943**	–	–	–	–	–	–	–	–
naive	0.945	0.954	0.959	0.868	0.841	0.916	0.935	0.924	0.924	0.932	0.813	0.887	0.907

Table S10-31. Comparison of different methods for state-level %ILI estimation in New Mexico. The MSE, MAE, and correlation are reported. The method with the best performance is highlighted in boldface for each metric in each period.

	Whole period	'14-'23	post-COVID	GFT period	'14-'15	'15-'16	'16-'17	'17-'18	'18-'19	'19-'20	'20-'21	'21-'22	'22-'23
RMSE													
ARGO-C	**0.585**	**0.575**	0.555	**0.665**	**0.702**	0.523	**0.990**	0.801	0.321	0.871	0.179	0.444	**1.103**
ARGOX	0.589	0.576	**0.551**	0.690	0.734	**0.520**	1.064	**0.719**	**0.294**	**0.839**	0.167	0.447	1.131
VAR1	1.260	1.593	2.074	1.217	1.310	0.886	1.402	1.417	1.764	3.741	1.135	1.597	1.939
GFT	–	–	–	0.860	0.916	–	–	–	–	–	–	–	–
naive	0.688	0.669	0.632	0.838	0.899	0.580	1.060	0.992	0.379	1.044	**0.150**	**0.430**	1.384
MAE													
ARGO-C	0.392	0.362	0.306	**0.502**	**0.517**	0.413	0.750	0.582	0.233	0.565	0.143	0.317	**0.866**
ARGOX	**0.390**	**0.360**	**0.304**	0.513	0.534	**0.401**	0.781	**0.546**	**0.232**	**0.552**	0.137	**0.315**	0.902
VAR1	1.010	1.145	1.394	0.858	0.942	0.694	1.161	1.002	1.690	2.529	1.079	1.114	1.763
GFT	–	–	–	0.651	0.696	–	–	–	–	–	–	–	–
naive	0.415	0.391	0.347	0.553	0.588	0.432	**0.732**	0.662	0.291	0.775	**0.119**	0.339	1.146
Correlation													
ARGO-C	**0.960**	**0.954**	0.937	**0.926**	**0.926**	**0.825**	**0.928**	0.963	0.956	0.936	0.758	**0.909**	0.802
ARGOX	0.959	0.954	**0.938**	0.922	0.920	0.822	0.916	**0.972**	**0.962**	**0.943**	0.769	0.898	0.791
VAR1	0.841	0.784	0.830	0.870	0.870	0.618	0.926	0.880	0.905	0.684	0.705	0.681	**0.867**
GFT	–	–	–	0.900	0.894	–	–	–	–	–	–	–	–
naive	0.945	0.939	0.921	0.886	0.878	0.794	0.915	0.942	0.940	0.901	**0.814**	0.867	0.702

Table S10-32. Comparison of different methods for state-level %ILI estimation in New York. The MSE, MAE, and correlation are reported. The method with the best performance is highlighted in boldface for each metric in each period.

	Whole period	'14-'23	post-COVID	GFT period	'14-'15	'15-'16	'16-'17	'17-'18	'18-'19	'19-'20	'20-'21	'21-'22	'22-'23
RMSE													
ARGO-C	**0.460**	**0.428**	**0.363**	**0.537**	**0.589**	**0.344**	**0.711**	**0.716**	0.335	0.372	0.121	**0.202**	**0.941**
ARGOX	0.492	0.458	0.387	0.696	0.765	0.359	0.726	0.739	**0.328**	**0.352**	0.112	0.205	1.012
VAR1	1.197	1.910	2.785	1.171	1.250	0.474	1.204	0.990	1.307	2.437	0.686	2.770	6.903
GFT	–	–	–	0.644	0.704	–	–	–	–	–	–	–	–
naive	0.693	0.617	0.442	1.022	1.125	0.456	0.917	1.065	0.541	0.634	**0.093**	0.324	1.078
MAE													
ARGO-C	**0.292**	**0.256**	**0.189**	**0.372**	**0.434**	**0.268**	**0.478**	**0.466**	0.210	0.277	0.091	0.166	**0.635**
ARGOX	0.297	0.262	0.197	0.421	0.497	0.269	0.495	0.500	**0.190**	**0.236**	0.085	**0.163**	0.725
VAR1	0.772	1.079	1.647	0.849	0.887	0.392	0.892	0.719	0.987	1.639	0.617	2.539	5.125
GFT	–	–	–	0.452	0.510	–	–	–	–	–	–	–	–
naive	0.409	0.343	0.221	0.509	0.606	0.348	0.680	0.725	0.374	0.454	**0.079**	0.204	0.781
Correlation													
ARGO-C	**0.967**	**0.966**	**0.964**	0.964	0.960	**0.937**	0.929	**0.962**	**0.983**	0.977	0.942	**0.971**	**0.870**
ARGOX	0.962	0.961	0.960	0.936	0.928	0.930	0.927	0.959	0.980	**0.979**	**0.950**	0.967	0.852
VAR1	0.795	0.670	0.690	0.823	0.796	0.897	**0.936**	0.919	0.759	0.848	0.674	0.646	0.163
GFT	–	–	–	**0.976**	**0.972**	–	–	–	–	–	–	–	–
naive	0.923	0.930	0.948	0.852	0.831	0.881	0.881	0.908	0.940	0.925	0.935	0.896	0.833

Table S10-33. Comparison of different methods for state-level %ILI estimation in North Carolina. The MSE, MAE, and correlation are reported. The method with the best performance is highlighted in boldface for each metric in each period.

	Whole period	'14-'23	post-COVID	GFT period	'14-'15	'15-'16	'16-'17	'17-'18	'18-'19	'19-'20	'20-'21	'21-'22	'22-'23
RMSE													
ARGO-C	**0.767**	**0.889**	**1.079**	**0.899**	**0.990**	**0.432**	**1.099**	0.776	**0.610**	**1.284**	**0.394**	**0.999**	**2.578**
ARGOX	0.786	0.911	1.106	0.902	0.992	0.435	1.114	0.796	0.648	1.335	0.396	1.061	2.631
VAR1	1.158	1.427	1.824	1.061	1.161	0.618	1.377	**0.729**	0.885	2.886	0.855	1.935	3.853
GFT	–	–	–	1.020	1.112	–	–	–	–	–	–	–	–
naive	0.891	1.011	1.202	0.960	1.056	0.495	1.342	0.988	0.775	1.480	0.412	1.077	2.861
MAE													
ARGO-C	**0.478**	**0.513**	**0.576**	**0.510**	**0.606**	**0.353**	**0.761**	0.596	**0.459**	**0.915**	**0.301**	**0.645**	**1.610**
ARGOX	0.491	0.526	0.591	0.512	0.608	0.357	0.776	0.622	0.489	0.966	0.301	0.686	1.644
VAR1	0.696	0.815	1.035	0.632	0.709	0.479	1.033	**0.585**	0.605	2.112	0.641	1.161	2.691
GFT	–	–	–	0.709	0.788	–	–	–	–	–	–	–	–
naive	0.556	0.603	0.690	0.557	0.663	0.405	0.899	0.720	0.604	1.128	0.340	0.799	1.949
Correlation													
ARGO-C	**0.875**	**0.868**	**0.847**	0.870	0.850	**0.739**	**0.670**	0.867	**0.881**	**0.835**	0.744	**0.877**	0.700
ARGOX	0.869	0.862	0.840	0.869	0.849	0.733	0.661	0.857	0.862	0.822	0.746	0.857	0.695
VAR1	0.726	0.672	0.570	**0.887**	**0.871**	0.580	0.567	**0.901**	0.753	0.331	-0.046	0.704	**0.804**
GFT	–	–	–	0.834	0.808	–	–	–	–	–	–	–	–
naive	0.839	0.840	0.826	0.859	0.839	0.687	0.575	0.748	0.814	0.793	**0.768**	0.859	0.675

Table S10-34. Comparison of different methods for state-level %ILI estimation in North Dakota. The MSE, MAE, and correlation are reported. The method with the best performance is highlighted in boldface for each metric in each period.

	Whole period	'14-'23	post-COVID	GFT period	'14-'15	'15-'16	'16-'17	'17-'18	'18-'19	'19-'20	'20-'21	'21-'22	'22-'23
RMSE													
ARGO-C	**0.301**	**0.343**	**0.410**	**0.517**	**0.568**	0.222	**0.235**	**0.347**	0.317	**0.416**	0.187	**0.407**	**0.888**
ARGOX	0.321	0.374	0.455	0.580	0.636	**0.214**	0.239	0.352	**0.315**	0.478	**0.187**	0.436	1.015
VAR1	1.528	1.458	1.320	1.907	2.055	0.733	2.348	2.549	0.952	2.274	0.248	0.911	2.513
GFT	–	–	–	0.954	1.039	–	–	–	–	–	–	–	–
naive	0.426	0.473	0.549	0.783	0.861	0.255	0.322	0.526	0.379	0.515	0.189	0.515	1.334
MAE													
ARGO-C	**0.185**	**0.210**	**0.256**	**0.243**	**0.278**	0.159	**0.175**	0.251	0.236	**0.307**	**0.138**	**0.335**	**0.631**
ARGOX	0.190	0.219	0.273	0.284	0.320	**0.148**	0.178	**0.244**	**0.227**	0.316	0.141	0.351	0.742
VAR1	0.994	0.892	0.702	1.424	1.548	0.570	1.763	1.807	0.758	1.571	0.200	0.752	1.910
GFT	–	–	–	0.774	0.870	–	–	–	–	–	–	–	–
naive	0.233	0.261	0.314	0.388	0.453	0.192	0.225	0.339	0.268	0.391	0.142	0.408	0.951
Correlation													
ARGO-C	**0.951**	**0.972**	**0.976**	0.925	0.920	0.866	**0.961**	**0.962**	0.899	**0.939**	0.750	**0.930**	**0.952**
ARGOX	0.944	0.966	0.970	0.903	0.896	**0.880**	0.959	0.961	**0.899**	0.918	**0.751**	0.916	0.934
VAR1	0.777	0.648	0.721	0.431	0.376	0.838	0.882	0.867	0.789	0.656	0.573	0.810	0.727
GFT	–	–	–	**0.971**	**0.969**	–	–	–	–	–	–	–	–
naive	0.903	0.946	0.957	0.826	0.811	0.835	0.922	0.910	0.860	0.899	0.744	0.878	0.887

Table S10-35. Comparison of different methods for state-level %ILI estimation in Ohio. The MSE, MAE, and correlation are reported. The method with the best performance is highlighted in boldface for each metric in each period.

	Whole period	'14-'23	post-COVID	GFT period	'14-'15	'15-'16	'16-'17	'17-'18	'18-'19	'19-'20	'20-'21	'21-'22	'22-'23
RMSE													
ARGO-C	0.955	0.901	**0.790**	1.444	1.576	0.890	1.552	0.853	**0.725**	**1.018**	0.579	1.014	**1.099**
ARGOX	**0.933**	**0.889**	0.801	1.435	1.564	0.887	1.500	**0.733**	0.747	1.068	**0.577**	**0.996**	1.121
VAR1	3.239	2.751	1.465	4.118	4.533	2.011	5.221	3.008	3.988	4.228	0.960	1.142	1.758
GFT	–	–	–	2.056	2.208	–	–	–	–	–	–	–	–
naive	0.987	0.925	0.796	**1.375**	**1.508**	**0.887**	**1.457**	1.015	0.994	1.176	0.618	1.002	1.130
MAE													
ARGO-C	0.601	0.573	**0.523**	0.982	**1.119**	0.726	1.087	0.598	**0.495**	**0.685**	**0.451**	0.656	**0.761**
ARGOX	**0.589**	**0.569**	0.532	0.996	1.126	0.724	**1.050**	**0.536**	0.508	0.707	0.456	**0.640**	0.816
VAR1	2.027	1.678	1.032	2.770	3.328	1.801	3.975	2.103	3.007	3.647	0.784	0.973	1.506
GFT	–	–	–	1.424	1.504	–	–	–	–	–	–	–	–
naive	0.650	0.609	0.533	**0.970**	1.123	**0.716**	1.091	0.763	0.706	0.812	0.500	0.692	0.916
Correlation													
ARGO-C	0.952	0.947	0.915	0.914	0.892	0.506	0.921	0.972	**0.973**	**0.941**	0.774	0.533	**0.915**
ARGOX	**0.955**	**0.949**	0.913	0.917	0.897	0.498	0.925	**0.978**	0.971	0.935	**0.777**	0.529	0.909
VAR1	0.709	0.491	0.709	0.541	0.479	0.257	0.743	0.765	0.513	0.864	0.571	0.431	0.869
GFT	–	–	–	0.842	0.809	–	–	–	–	–	–	–	–
naive	0.950	0.946	**0.919**	**0.923**	**0.904**	**0.535**	**0.931**	0.941	0.940	0.921	0.751	**0.534**	0.898

Table S10-36. Comparison of different methods for state-level %ILI estimation in Oklahoma. The MSE, MAE, and correlation are reported. The method with the best performance is highlighted in boldface for each metric in each period.

	Whole period	’14-’23	post-COVID	GFT period	’14-’15	’15-’16	’16-’17	’17-’18	’18-’19	’19-’20	’20-’21	’21-’22	’22-’23
RMSE													
ARGO-C	0.428	0.397	0.332	0.371	0.402	**0.569**	0.817	0.339	**0.358**	0.413	0.158	0.222	**0.775**
ARGOX	**0.425**	**0.393**	**0.324**	0.368	0.399	0.579	**0.810**	**0.290**	0.367	**0.387**	0.144	**0.197**	0.804
VAR1	2.425	2.061	1.102	3.854	4.239	1.258	3.884	2.004	2.512	2.624	0.986	1.147	1.567
GFT	–	–	–	0.570	0.487	–	–	–	–	–	–	–	–
naive	0.500	0.478	0.433	**0.323**	**0.348**	0.632	0.946	0.442	0.450	0.757	**0.127**	0.283	0.891
MAE													
ARGO-C	0.260	0.237	0.195	0.275	0.306	**0.419**	0.465	0.231	**0.253**	**0.296**	0.120	0.177	**0.580**
ARGOX	**0.258**	**0.234**	**0.188**	0.273	0.303	0.429	**0.464**	**0.205**	0.265	0.297	0.114	**0.161**	0.589
VAR1	1.527	1.253	0.745	2.792	3.300	1.129	2.993	1.324	2.038	2.044	0.781	0.927	1.275
GFT	–	–	–	0.473	0.396	–	–	–	–	–	–	–	–
naive	0.292	0.273	0.238	**0.254**	**0.280**	0.428	0.560	0.319	0.275	0.580	**0.097**	0.189	0.672
Correlation													
ARGO-C	0.940	0.945	0.959	0.794	0.726	**0.741**	0.781	0.983	**0.953**	0.969	0.938	0.919	**0.900**
ARGOX	**0.940**	**0.946**	**0.960**	0.795	0.724	0.731	**0.781**	**0.986**	0.952	**0.973**	**0.945**	**0.936**	0.889
VAR1	0.727	0.720	0.823	0.593	0.469	0.658	0.579	0.917	0.856	0.713	0.533	0.405	0.763
GFT	–	–	–	0.755	**0.822**	–	–	–	–	–	–	–	–
naive	0.918	0.922	0.931	**0.799**	0.737	0.713	0.728	0.950	0.919	0.892	0.939	0.874	0.867

Table S10-37. Comparison of different methods for state-level %ILI estimation in Oregon. The MSE, MAE, and correlation are reported. The method with the best performance is highlighted in boldface for each metric in each period.

	Whole period	’14-’23	post-COVID	GFT period	’14-’15	’15-’16	’16-’17	’17-’18	’18-’19	’19-’20	’20-’21	’21-’22	’22-’23
RMSE													
ARGO-C	**0.328**	**0.306**	0.262	**0.492**	**0.529**	0.341	**0.431**	0.413	0.190	0.442	0.143	**0.261**	**0.322**
ARGOX	0.336	0.309	**0.250**	0.535	0.575	**0.332**	0.456	**0.388**	**0.190**	**0.404**	**0.131**	0.264	0.352
VAR1	1.117	0.962	0.574	2.077	2.247	1.147	1.112	1.065	0.706	0.878	0.209	0.670	1.090
GFT	–	–	–	0.589	0.640	–	–	–	–	–	–	–	–
naive	0.465	0.429	0.354	0.764	0.832	0.454	0.564	0.573	0.286	0.673	0.138	0.340	0.498
MAE													
ARGO-C	**0.216**	0.193	0.151	**0.340**	**0.365**	0.250	**0.314**	0.267	**0.146**	0.241	0.105	0.183	**0.241**
ARGOX	0.216	**0.191**	**0.145**	0.366	0.393	**0.241**	0.329	**0.235**	0.153	**0.235**	**0.097**	**0.169**	0.259
VAR1	0.751	0.626	0.392	1.605	1.757	1.019	0.859	0.587	0.546	0.660	0.168	0.491	0.981
GFT	–	–	–	0.480	0.535	–	–	–	–	–	–	–	–
naive	0.288	0.256	0.197	0.504	0.565	0.336	0.396	0.368	0.212	0.500	0.101	0.211	0.376
Correlation													
ARGO-C	**0.969**	**0.969**	0.967	**0.951**	**0.949**	0.871	**0.940**	0.977	0.977	0.961	0.917	**0.965**	**0.958**
ARGOX	0.968	0.969	**0.971**	0.943	0.940	**0.877**	0.932	**0.982**	**0.978**	**0.969**	**0.926**	0.956	0.948
VAR1	0.707	0.719	0.871	0.714	0.678	0.669	0.705	0.808	0.746	0.845	0.824	0.412	0.805
GFT	–	–	–	0.946	0.942	–	–	–	–	–	–	–	–
naive	0.937	0.940	0.943	0.880	0.867	0.793	0.900	0.944	0.943	0.904	0.915	0.869	0.892

Table S10-38. Comparison of different methods for state-level %ILI estimation in Pennsylvania. The MSE, MAE, and correlation are reported. The method with the best performance is highlighted in boldface for each metric in each period.

	Whole period	’14-’23	post-COVID	GFT period	’14-’15	’15-’16	’16-’17	’17-’18	’18-’19	’19-’20	’20-’21	’21-’22	’22-’23
RMSE													
ARGO-C	**0.445**	**0.473**	0.522	**0.475**	**0.522**	**0.225**	**0.471**	0.639	**0.509**	**0.911**	0.149	0.595	0.899
ARGOX	0.457	0.488	0.539	0.483	0.531	0.232	0.486	**0.638**	0.520	1.003	0.144	0.579	**0.897**
VAR1	1.227	1.181	1.090	1.614	1.707	1.538	0.914	0.907	1.226	2.481	0.523	0.790	1.319
GFT	–	–	–	0.674	0.709	–	–	–	–	–	–	–	–
naive	0.542	0.528	**0.501**	0.475	0.522	0.239	0.542	0.825	0.654	0.959	**0.135**	**0.577**	1.051
MAE													
ARGO-C	**0.260**	**0.265**	0.276	**0.261**	**0.305**	0.164	**0.315**	0.468	**0.362**	**0.556**	0.121	0.409	**0.595**
ARGOX	0.266	0.270	0.279	0.270	0.313	0.169	0.325	**0.465**	0.372	0.607	0.118	0.396	0.625
VAR1	0.976	0.877	0.693	1.376	1.445	1.480	0.785	0.696	0.913	1.910	0.466	0.596	1.021
GFT	–	–	–	0.593	0.619	–	–	–	–	–	–	–	–
naive	0.304	0.289	**0.261**	0.285	0.335	**0.153**	0.365	0.578	0.490	0.618	**0.105**	**0.376**	0.725
Correlation													
ARGO-C	**0.969**	**0.954**	0.906	0.916	0.906	**0.920**	**0.935**	0.952	**0.963**	**0.950**	0.776	**0.626**	0.928
ARGOX	0.967	0.951	0.900	0.914	0.905	0.913	0.928	**0.953**	0.960	0.940	**0.802**	0.626	0.928
VAR1	0.787	0.746	0.630	0.763	0.735	0.767	0.890	0.900	0.867	0.549	0.702	0.333	**0.933**
GFT	–	–	–	**0.943**	**0.937**	–	–	–	–	–	–	–	–
naive	0.951	0.944	**0.919**	0.911	0.901	0.907	0.904	0.915	0.936	0.946	0.746	0.589	0.890

Table S10-39. Comparison of different methods for state-level %ILI estimation in Rhode Island. The MSE, MAE, and correlation are reported. The method with the best performance is highlighted in boldface for each metric in each period.

	Whole period	'14-'23	post-COVID	GFT period	'14-'15	'15-'16	'16-'17	'17-'18	'18-'19	'19-'20	'20-'21	'21-'22	'22-'23
RMSE													
ARGO-C	**0.567**	**0.583**	**0.612**	**0.512**	**0.560**	0.332	**0.913**	**0.679**	0.689	**0.790**	0.338	**0.316**	**1.498**
ARGOX	0.594	0.620	0.665	0.593	0.648	**0.330**	0.942	0.709	**0.682**	0.858	0.320	0.319	1.683
VAR1	2.333	2.324	2.308	1.559	1.715	0.799	2.941	4.113	2.165	4.152	1.123	1.449	5.240
GFT	–	–	–	1.584	1.725	–	–	–	–	–	–	–	–
naive	0.875	0.841	0.775	0.796	0.873	0.376	1.006	1.358	0.933	1.589	**0.246**	0.503	1.788
MAE													
ARGO-C	**0.347**	**0.352**	**0.362**	**0.265**	**0.301**	**0.258**	**0.733**	**0.447**	**0.433**	**0.578**	0.270	0.238	**1.081**
ARGOX	0.363	0.364	0.366	0.284	0.317	0.261	0.763	0.457	0.450	0.622	0.243	**0.222**	1.258
VAR1	1.471	1.486	1.513	0.834	0.989	0.633	2.505	3.045	1.807	3.584	0.987	1.136	3.904
GFT	–	–	–	1.319	1.483	–	–	–	–	–	–	–	–
naive	0.514	0.481	0.420	0.421	0.481	0.292	0.795	0.903	0.683	1.174	**0.195**	0.342	1.365
Correlation													
ARGO-C	**0.980**	**0.975**	**0.961**	0.944	0.939	0.855	**0.913**	**0.985**	**0.961**	**0.970**	0.920	**0.979**	**0.849**
ARGOX	0.978	0.972	0.955	0.919	0.910	**0.856**	0.906	0.984	0.958	0.965	**0.931**	0.971	0.813
VAR1	0.782	0.737	0.596	0.295	0.208	0.600	0.711	0.751	0.840	0.753	0.758	0.563	-0.169
GFT	–	–	–	**0.977**	**0.975**	–	–	–	–	–	–	–	–
naive	0.952	0.949	0.939	0.851	0.834	0.823	0.888	0.939	0.915	0.878	0.930	0.905	0.791

Table S10-40. Comparison of different methods for state-level %ILI estimation in South Carolina. The MSE, MAE, and correlation are reported. The method with the best performance is highlighted in boldface for each metric in each period.

	Whole period	'14-'23	post-COVID	GFT period	'14-'15	'15-'16	'16-'17	'17-'18	'18-'19	'19-'20	'20-'21	'21-'22	'22-'23
RMSE													
ARGO-C	**0.326**	**0.306**	**0.265**	**0.315**	**0.329**	**0.292**	**0.418**	**0.492**	**0.358**	**0.355**	0.182	**0.283**	**0.430**
ARGOX	0.329	0.310	0.270	0.318	0.332	0.295	0.425	0.495	0.362	0.368	**0.179**	0.288	0.443
VAR1	2.064	2.162	2.333	0.832	0.888	0.722	1.882	3.319	2.914	4.225	1.169	1.176	5.356
GFT	–	–	–	1.033	1.132	–	–	–	–	–	–	–	–
naive	0.354	0.330	0.282	0.362	0.380	0.309	0.427	0.542	0.376	0.403	0.184	0.302	0.468
MAE													
ARGO-C	**0.233**	**0.215**	**0.183**	**0.235**	**0.243**	0.218	**0.297**	**0.369**	**0.279**	**0.233**	0.148	**0.204**	**0.336**
ARGOX	0.235	0.217	0.184	0.238	0.245	**0.217**	0.299	0.371	0.283	0.247	**0.147**	0.209	0.350
VAR1	1.264	1.352	1.514	0.588	0.615	0.559	1.481	2.117	2.444	3.700	0.978	0.965	4.631
GFT	–	–	–	0.512	0.584	–	–	–	–	–	–	–	–
naive	0.257	0.233	0.188	0.263	0.270	0.244	0.318	0.428	0.284	0.257	0.154	0.226	0.367
Correlation													
ARGO-C	**0.950**	**0.950**	**0.937**	**0.921**	**0.912**	**0.851**	**0.925**	**0.923**	**0.876**	**0.960**	**0.381**	**0.907**	**0.905**
ARGOX	0.949	0.949	0.933	0.919	0.910	0.847	0.923	0.922	0.873	0.957	0.380	0.902	0.898
VAR1	0.812	0.750	0.702	0.806	0.780	0.434	0.759	0.906	0.851	0.849	-0.145	0.803	0.359
GFT	–	–	–	0.898	0.886	–	–	–	–	–	–	–	–
naive	0.941	0.941	0.925	0.898	0.885	0.840	0.924	0.903	0.844	0.948	0.378	0.893	0.880

Table S10-41. Comparison of different methods for state-level %ILI estimation in South Dakota. The MSE, MAE, and correlation are reported. The method with the best performance is highlighted in boldface for each metric in each period.

	Whole period	'14-'23	post-COVID	GFT period	'14-'15	'15-'16	'16-'17	'17-'18	'18-'19	'19-'20	'20-'21	'21-'22	'22-'23
RMSE													
ARGO-C	**0.703**	**0.683**	**0.644**	**0.572**	**0.620**	**0.478**	0.883	1.061	**0.467**	**1.195**	0.232	0.400	**1.620**
ARGOX	0.728	0.718	0.698	0.591	0.641	0.482	**0.870**	**1.026**	0.505	1.369	0.222	**0.368**	1.775
VAR1	1.943	2.166	2.529	1.593	1.736	0.667	1.983	1.858	1.331	4.675	0.810	1.494	6.530
GFT	–	–	–	1.239	1.341	–	–	–	–	–	–	–	–
naive	0.866	0.820	0.727	0.890	0.974	0.507	0.991	1.212	0.707	1.546	**0.126**	0.512	1.837
MAE													
ARGO-C	**0.472**	**0.429**	**0.350**	0.410	0.453	0.391	0.684	0.670	**0.360**	**0.896**	0.176	0.249	**1.273**
ARGOX	0.477	0.438	0.365	**0.407**	**0.451**	0.394	**0.663**	**0.651**	0.366	0.981	0.171	**0.249**	1.429
VAR1	1.145	1.311	1.618	0.970	1.078	0.528	1.746	1.462	0.940	3.731	0.743	1.289	5.572
GFT	–	–	–	0.990	1.089	–	–	–	–	–	–	–	–
naive	0.507	0.464	0.385	0.534	0.612	**0.358**	0.711	0.726	0.522	1.055	**0.103**	0.362	1.494
Correlation													
ARGO-C	**0.951**	**0.954**	**0.961**	0.954	0.950	0.844	0.857	0.907	**0.950**	**0.944**	0.914	**0.972**	**0.891**
ARGOX	0.947	0.950	0.955	0.952	0.948	**0.844**	0.862	**0.915**	0.936	0.926	0.918	0.968	0.870
VAR1	0.658	0.604	0.672	0.695	0.656	0.770	**0.887**	0.631	0.732	0.725	0.350	0.823	0.225
GFT	–	–	–	**0.966**	**0.964**	–	–	–	–	–	–	–	–
naive	0.927	0.936	0.951	0.891	0.879	0.836	0.822	0.866	0.869	0.898	**0.932**	0.902	0.861

Table S10-42. Comparison of different methods for state-level %ILI estimation in Tennessee. The MSE, MAE, and correlation are reported. The method with the best performance is highlighted in boldface for each metric in each period.

	Whole period	’14-’23	post-COVID	GFT period	’14-’15	’15-’16	’16-’17	’17-’18	’18-’19	’19-’20	’20-’21	’21-’22	’22-’23
RMSE													
ARGO-C	**0.851**	**0.751**	**0.520**	1.473	1.609	0.564	**0.761**	1.087	0.647	**1.147**	0.342	0.386	**0.816**
ARGOX	0.852	0.763	0.565	1.466	1.603	**0.552**	0.792	**1.049**	**0.623**	1.301	0.347	**0.360**	0.840
VAR1	2.766	2.323	1.106	3.051	3.298	2.776	1.873	4.760	3.334	2.524	0.391	0.889	2.359
GFT	–	–	–	**1.253**	**1.368**	–	–	–	–	–	–	–	–
naive	0.996	0.871	0.570	1.544	1.690	0.577	0.796	1.398	0.822	1.536	**0.338**	0.555	0.841
MAE													
ARGO-C	0.509	**0.447**	**0.331**	0.848	0.953	0.451	**0.574**	**0.664**	0.443	**0.833**	0.264	**0.270**	**0.660**
ARGOX	**0.509**	0.448	0.336	0.870	0.989	**0.448**	0.590	0.671	**0.415**	0.868	0.256	0.271	0.715
VAR1	2.134	1.637	0.717	2.567	2.819	2.411	1.319	3.934	2.974	2.063	0.276	0.736	1.752
GFT	–	–	–	**0.835**	**0.951**	–	–	–	–	–	–	–	–
naive	0.595	0.513	0.359	0.893	1.016	0.454	0.577	0.957	0.634	1.100	**0.218**	0.378	0.690
Correlation													
ARGO-C	**0.961**	**0.963**	**0.967**	0.860	0.837	0.824	**0.953**	0.970	0.973	**0.955**	0.568	0.928	**0.923**
ARGOX	0.961	0.962	0.962	0.861	0.837	**0.830**	0.949	**0.972**	**0.975**	0.942	**0.571**	**0.937**	0.918
VAR1	0.820	0.763	0.902	0.848	0.831	-0.195	0.799	0.886	0.893	0.836	0.528	0.887	0.876
GFT	–	–	–	**0.928**	**0.916**	–	–	–	–	–	–	–	–
naive	0.947	0.951	0.958	0.854	0.832	0.816	0.945	0.949	0.946	0.914	0.513	0.854	0.909

Table S10-43. Comparison of different methods for state-level %ILI estimation in Texas. The MSE, MAE, and correlation are reported. The method with the best performance is highlighted in boldface for each metric in each period.

	Whole period	’14-’23	post-COVID	GFT period	’14-’15	’15-’16	’16-’17	’17-’18	’18-’19	’19-’20	’20-’21	’21-’22	’22-’23
RMSE													
ARGO-C	**0.430**	**0.410**	**0.372**	**0.296**	**0.312**	**0.330**	**0.413**	0.397	**0.619**	**0.803**	0.181	0.355	**0.398**
ARGOX	0.445	0.425	0.386	0.300	0.317	0.375	0.449	**0.392**	0.627	0.857	0.170	**0.338**	0.458
VAR1	3.032	2.584	1.417	2.404	2.607	1.991	3.754	6.236	1.856	3.097	0.660	0.830	3.268
GFT	–	–	–	1.083	1.163	–	–	–	–	–	–	–	–
naive	0.507	0.489	0.454	0.512	0.550	0.429	0.439	0.451	0.658	0.997	**0.154**	0.371	0.695
MAE													
ARGO-C	**0.296**	**0.280**	0.251	**0.228**	**0.240**	**0.263**	0.293	0.301	**0.474**	**0.583**	0.148	0.277	**0.315**
ARGOX	0.302	0.282	**0.246**	0.233	0.244	0.289	0.307	**0.296**	0.477	0.593	0.135	**0.260**	0.348
VAR1	2.105	1.703	0.957	1.795	1.977	1.850	2.877	5.066	1.532	2.543	0.583	0.503	2.991
GFT	–	–	–	1.002	1.106	–	–	–	–	–	–	–	–
naive	0.334	0.313	0.273	0.355	0.381	0.329	**0.289**	0.304	0.515	0.724	**0.124**	0.291	0.517
Correlation													
ARGO-C	**0.962**	**0.960**	**0.957**	**0.970**	**0.965**	**0.930**	**0.909**	0.891	**0.938**	**0.938**	0.601	**0.884**	**0.971**
ARGOX	0.959	0.957	0.955	0.970	0.965	0.909	0.889	**0.893**	0.935	0.930	**0.617**	0.876	0.957
VAR1	0.693	0.702	0.842	0.920	0.914	0.651	0.048	0.853	0.934	0.906	0.335	0.531	0.734
GFT	–	–	–	0.951	0.957	–	–	–	–	–	–	–	–
naive	0.947	0.945	0.937	0.911	0.894	0.885	0.889	0.868	0.921	0.899	0.541	0.809	0.894

Table S10-44. Comparison of different methods for state-level %ILI estimation in Utah. The MSE, MAE, and correlation are reported. The method with the best performance is highlighted in boldface for each metric in each period.

	Whole period	’14-’23	post-COVID	GFT period	’14-’15	’15-’16	’16-’17	’17-’18	’18-’19	’19-’20	’20-’21	’21-’22	’22-’23
RMSE													
ARGO-C	0.493	**0.514**	0.550	**0.753**	0.810	0.430	0.325	0.537	0.540	**0.545**	**0.230**	0.246	1.456
ARGOX	**0.493**	0.515	0.554	0.754	**0.809**	**0.429**	**0.320**	**0.533**	**0.539**	0.555	0.232	0.248	1.468
VAR1	1.101	1.103	1.106	1.289	1.355	0.591	0.959	1.417	1.214	1.463	0.335	1.746	1.350
GFT	–	–	–	1.220	1.330	–	–	–	–	–	–	–	–
naive	0.536	0.531	**0.520**	0.820	0.896	0.469	0.357	0.579	0.590	0.619	0.246	**0.224**	**1.325**
MAE													
ARGO-C	**0.348**	**0.320**	0.269	**0.517**	0.557	**0.324**	0.257	**0.405**	**0.406**	**0.387**	**0.174**	0.170	0.910
ARGOX	0.348	0.320	**0.268**	0.518	**0.553**	0.324	**0.254**	0.406	0.409	0.389	0.175	0.168	0.915
VAR1	0.854	0.867	0.891	0.908	0.907	0.488	0.781	1.166	1.008	1.167	0.292	1.685	1.210
GFT	–	–	–	0.833	0.938	–	–	–	–	–	–	–	–
naive	0.381	0.342	0.271	0.560	0.639	0.360	0.264	0.450	0.447	0.445	0.187	**0.165**	**0.845**
Correlation													
ARGO-C	**0.914**	**0.902**	0.863	0.904	0.872	0.790	0.797	0.920	0.878	**0.934**	**0.716**	**0.773**	0.691
ARGOX	0.914	0.901	0.863	**0.904**	**0.872**	0.788	**0.798**	**0.921**	**0.878**	0.932	0.712	0.763	0.691
VAR1	0.618	0.616	0.730	0.763	0.693	**0.811**	0.075	0.834	0.745	0.867	0.594	0.560	**0.801**
GFT	–	–	–	0.836	0.814	–	–	–	–	–	–	–	–
naive	0.901	0.897	**0.873**	0.884	0.849	0.753	0.754	0.910	0.859	0.918	0.691	0.743	0.717

Table S10-45. Comparison of different methods for state-level %ILI estimation in Vermont. The MSE, MAE, and correlation are reported. The method with the best performance is highlighted in boldface for each metric in each period.

	Whole period	’14-’23	post-COVID	GFT period	’14-’15	’15-’16	’16-’17	’17-’18	’18-’19	’19-’20	’20-’21	’21-’22	’22-’23
RMSE													
ARGO-C	**0.418**	**0.439**	**0.475**	**0.623**	**0.684**	0.251	**0.476**	0.658	0.431	**0.364**	0.149	**0.483**	**0.885**
ARGOX	0.431	0.467	0.528	0.682	0.748	**0.233**	0.495	**0.636**	**0.418**	0.469	0.136	0.596	0.984
VAR1	0.638	0.761	0.947	0.933	1.026	0.289	0.688	1.023	0.565	0.926	**0.118**	0.938	2.222
GFT	–	–	–	1.045	1.147	–	–	–	–	–	–	–	–
naive	0.628	0.636	0.650	0.947	1.042	0.290	0.646	0.990	0.561	0.914	0.120	0.849	1.050
MAE													
ARGO-C	**0.238**	**0.240**	**0.243**	**0.300**	**0.349**	0.189	**0.319**	0.443	0.292	**0.266**	0.124	**0.269**	**0.682**
ARGOX	0.243	0.254	0.273	0.328	0.377	**0.172**	0.331	**0.416**	**0.286**	0.337	0.113	0.345	0.776
VAR1	0.330	0.377	0.464	0.437	0.510	0.232	0.396	0.624	0.416	0.667	**0.097**	0.542	1.722
GFT	–	–	–	0.519	0.593	–	–	–	–	–	–	–	–
naive	0.332	0.333	0.335	0.466	0.547	0.240	0.381	0.613	0.411	0.674	0.098	0.497	0.837
Correlation													
ARGO-C	**0.980**	**0.976**	**0.971**	0.957	0.953	0.918	**0.942**	0.980	**0.978**	**0.991**	0.888	**0.945**	**0.927**
ARGOX	0.979	0.973	0.965	0.945	0.939	**0.936**	0.936	**0.981**	0.977	0.985	0.908	0.915	0.912
VAR1	0.951	0.928	0.895	0.885	0.872	0.887	0.871	0.939	0.954	0.930	**0.921**	0.816	0.776
GFT	–	–	–	**0.976**	**0.974**	–	–	–	–	–	–	–	–
naive	0.953	0.950	0.946	0.886	0.873	0.889	0.885	0.939	0.956	0.933	0.920	0.836	0.884

Table S10-46. Comparison of different methods for state-level %ILI estimation in Virginia. The MSE, MAE, and correlation are reported. The method with the best performance is highlighted in boldface for each metric in each period.

	Whole period	’14-’23	post-COVID	GFT period	’14-’15	’15-’16	’16-’17	’17-’18	’18-’19	’19-’20	’20-’21	’21-’22	’22-’23
RMSE													
ARGO-C	**0.420**	**0.487**	**0.590**	0.347	0.380	0.323	**0.527**	**0.658**	**0.428**	**0.602**	0.149	0.298	**1.650**
ARGOX	0.433	0.516	0.643	**0.347**	**0.379**	**0.315**	0.568	0.663	0.443	0.733	0.139	**0.252**	1.762
VAR1	1.241	1.477	1.837	1.384	1.517	0.701	1.061	1.381	0.992	2.428	0.267	1.054	4.934
GFT	–	–	–	0.784	0.840	–	–	–	–	–	–	–	–
naive	0.494	0.583	0.721	0.393	0.430	0.338	0.568	0.753	0.549	0.976	**0.122**	0.294	1.896
MAE													
ARGO-C	**0.258**	**0.256**	**0.254**	**0.233**	**0.263**	0.267	**0.347**	0.370	**0.277**	**0.454**	0.112	0.237	**1.025**
ARGOX	0.258	0.260	0.263	0.236	0.267	**0.262**	0.365	**0.352**	0.286	0.510	0.103	**0.204**	1.097
VAR1	0.907	0.901	0.890	1.043	1.207	0.637	0.827	1.039	0.896	1.799	0.203	0.915	3.355
GFT	–	–	–	0.695	0.759	–	–	–	–	–	–	–	–
naive	0.295	0.302	0.315	0.284	0.325	0.283	0.352	0.422	0.358	0.697	**0.099**	0.211	1.384
Correlation													
ARGO-C	**0.950**	**0.955**	**0.956**	0.939	0.933	0.824	**0.845**	**0.877**	**0.925**	**0.965**	0.642	0.874	**0.913**
ARGOX	0.947	0.949	0.948	0.940	0.934	**0.831**	0.817	0.876	0.920	0.951	**0.661**	**0.910**	0.897
VAR1	0.521	0.454	0.518	0.617	0.549	0.604	0.012	0.373	0.731	0.362	0.557	0.508	0.225
GFT	–	–	–	**0.961**	**0.959**	–	–	–	–	–	–	–	–
naive	0.932	0.936	0.936	0.924	0.915	0.809	0.834	0.847	0.876	0.903	0.621	0.881	0.884

Table S10-47. Comparison of different methods for state-level %ILI estimation in Washington. The MSE, MAE, and correlation are reported. The method with the best performance is highlighted in boldface for each metric in each period.

	Whole period	’14-’23	post-COVID	GFT period	’14-’15	’15-’16	’16-’17	’17-’18	’18-’19	’19-’20	’20-’21	’21-’22	’22-’23
RMSE													
ARGO-C	**0.499**	**0.504**	**0.512**	**0.621**	**0.678**	0.367	0.399	**0.818**	0.627	**0.678**	0.393	0.461	**0.744**
ARGOX	0.519	0.523	0.530	0.633	0.690	**0.357**	**0.389**	0.870	**0.614**	0.815	**0.384**	0.447	0.760
VAR1	1.344	1.442	1.607	1.742	1.916	0.963	1.270	2.004	1.517	1.859	0.387	0.815	4.273
GFT	–	–	–	1.002	1.004	–	–	–	–	–	–	–	–
naive	0.645	0.611	0.543	0.765	0.838	0.391	0.426	1.117	0.692	0.968	0.435	**0.431**	0.836
MAE													
ARGO-C	**0.312**	**0.319**	**0.331**	0.412	0.459	0.309	0.331	**0.509**	**0.406**	**0.396**	0.232	0.360	**0.609**
ARGOX	0.320	0.325	0.333	**0.405**	**0.450**	0.295	0.316	0.548	0.419	0.487	0.223	0.332	0.625
VAR1	0.843	0.832	0.811	1.151	1.360	0.868	0.902	1.318	1.118	1.453	**0.210**	0.647	2.912
GFT	–	–	–	0.915	0.900	–	–	–	–	–	–	–	–
naive	0.356	0.352	0.346	0.491	0.563	**0.281**	**0.298**	0.665	0.437	0.659	0.261	**0.285**	0.703
Correlation													
ARGO-C	**0.958**	**0.946**	**0.892**	0.958	0.953	0.798	0.931	**0.931**	0.923	**0.955**	0.368	0.333	**0.779**
ARGOX	0.954	0.941	0.887	0.956	0.951	**0.811**	**0.935**	0.922	**0.927**	0.934	**0.403**	0.297	0.771
VAR1	0.737	0.595	0.723	0.900	0.886	0.728	0.455	0.748	0.695	0.630	0.245	-0.214	0.627
GFT	–	–	–	**0.966**	**0.961**	–	–	–	–	–	–	–	–
naive	0.930	0.922	0.884	0.930	0.919	0.798	0.921	0.872	0.907	0.910	0.317	**0.396**	0.712

Table S10-48. Comparison of different methods for state-level %ILI estimation in West Virginia. The MSE, MAE, and correlation are reported. The method with the best performance is highlighted in boldface for each metric in each period.

	Whole period	’14-’23	post-COVID	GFT period	’14-’15	’15-’16	’16-’17	’17-’18	’18-’19	’19-’20	’20-’21	’21-’22	’22-’23
RMSE													
ARGO-C	**0.371**	0.357	0.329	**0.529**	**0.562**	0.333	0.396	0.385	0.270	0.410	0.126	0.209	**0.394**
ARGOX	0.372	**0.356**	**0.324**	0.555	0.596	**0.330**	**0.380**	**0.354**	**0.267**	**0.405**	0.131	**0.203**	0.404
VAR1	0.474	0.445	0.386	0.759	0.827	0.351	0.447	0.552	0.297	0.616	0.089	0.232	0.505
GFT	–	–	–	0.784	0.842	–	–	–	–	–	–	–	–
naive	0.446	0.428	0.392	0.717	0.781	0.347	0.424	0.459	0.294	0.575	**0.085**	0.236	0.486
MAE													
ARGO-C	0.273	0.243	0.186	**0.407**	**0.430**	0.278	0.322	0.295	0.212	0.346	0.097	0.169	**0.312**
ARGOX	**0.271**	**0.239**	**0.181**	0.415	0.445	**0.268**	**0.319**	**0.269**	**0.211**	**0.334**	0.103	**0.160**	0.314
VAR1	0.322	0.278	0.196	0.467	0.519	0.299	0.360	0.415	0.237	0.461	0.073	0.171	0.409
GFT	–	–	–	0.635	0.686	–	–	–	–	–	–	–	–
naive	0.305	0.266	0.192	0.461	0.517	0.298	0.344	0.353	0.224	0.414	**0.067**	0.177	0.383
Correlation													
ARGO-C	**0.953**	0.954	0.954	**0.935**	0.909	0.737	0.887	0.947	0.894	0.965	0.919	0.896	**0.939**
ARGOX	0.953	**0.954**	**0.955**	0.929	0.896	**0.745**	**0.895**	**0.956**	**0.897**	**0.969**	0.913	**0.897**	0.934
VAR1	0.931	0.931	0.937	0.881	0.816	0.738	0.866	0.915	0.880	0.917	0.931	0.798	0.903
GFT	–	–	–	0.896	**0.914**	–	–	–	–	–	–	–	–
naive	0.933	0.936	0.938	0.883	0.822	0.743	0.869	0.921	0.885	0.923	**0.932**	0.799	0.905

Table S10-49. Comparison of different methods for state-level %ILI estimation in Wisconsin. The MSE, MAE, and correlation are reported. The method with the best performance is highlighted in boldface for each metric in each period.

	Whole period	’14-’23	post-COVID	GFT period	’14-’15	’15-’16	’16-’17	’17-’18	’18-’19	’19-’20	’20-’21	’21-’22	’22-’23
RMSE													
ARGO-C	**0.487**	0.613	0.795	0.357	0.392	**0.348**	**0.533**	0.728	**0.735**	**0.773**	0.254	0.795	1.812
ARGOX	0.493	**0.607**	**0.776**	**0.342**	**0.374**	0.348	0.558	**0.695**	0.745	0.793	**0.240**	**0.763**	**1.792**
VAR1	0.909	0.994	1.134	1.372	1.500	0.779	0.860	1.139	0.815	1.230	0.376	1.416	2.262
GFT	–	–	–	0.588	0.596	–	–	–	–	–	–	–	–
naive	0.551	0.685	0.881	0.357	0.391	0.373	0.582	0.810	0.825	0.929	0.256	0.802	2.118
MAE													
ARGO-C	**0.324**	0.374	0.468	0.280	0.327	**0.233**	**0.390**	0.509	0.559	**0.575**	0.184	0.582	1.307
ARGOX	0.326	**0.373**	**0.460**	0.275	0.318	0.237	0.416	**0.490**	**0.558**	0.602	**0.175**	**0.565**	**1.307**
VAR1	0.644	0.666	0.707	0.917	1.031	0.652	0.668	0.912	0.623	0.883	0.235	1.042	1.739
GFT	–	–	–	0.503	0.496	–	–	–	–	–	–	–	–
naive	0.353	0.411	0.520	**0.266**	**0.310**	0.265	0.448	0.601	0.593	0.696	0.199	0.566	1.653
Correlation													
ARGO-C	**0.956**	0.926	0.866	0.939	0.918	0.891	**0.862**	0.954	**0.911**	**0.913**	0.281	0.696	0.724
ARGOX	0.955	**0.928**	**0.873**	**0.945**	**0.927**	**0.891**	0.850	**0.959**	0.908	0.908	**0.297**	0.722	0.727
VAR1	0.847	0.796	0.744	0.900	0.886	0.710	0.632	0.912	0.888	0.810	0.241	0.517	**0.882**
GFT	–	–	–	0.877	0.856	–	–	–	–	–	–	–	–
naive	0.943	0.911	0.847	0.940	0.921	0.881	0.842	0.938	0.890	0.871	0.159	**0.731**	0.656

Table S10-50. Comparison of different methods for state-level %ILI estimation in Wyoming. The MSE, MAE, and correlation are reported. The method with the best performance is highlighted in boldface for each metric in each period.

	Whole period	’14-’23	post-COVID	GFT period	’14-’15	’15-’16	’16-’17	’17-’18	’18-’19	’19-’20	’20-’21	’21-’22	’22-’23
RMSE													
ARGO-C	**0.296**	**0.520**	**0.780**	0.205	0.217	**0.196**	**0.357**	**0.516**	**0.196**	1.406	0.192	**0.571**	**1.128**
ARGOX	0.308	0.527	0.786	**0.202**	**0.215**	0.204	0.392	0.528	0.214	**1.397**	0.165	0.596	1.186
VAR1	1.284	1.751	2.386	0.941	0.910	1.384	1.391	1.102	1.524	3.637	0.386	1.543	4.954
GFT	–	–	–	0.531	0.554	–	–	–	–	–	–	–	–
naive	0.406	0.609	0.868	0.231	0.243	0.262	0.393	0.773	0.324	1.585	**0.085**	0.641	1.338
MAE													
ARGO-C	**0.197**	**0.255**	0.361	0.163	0.173	**0.163**	**0.268**	0.359	**0.165**	**0.751**	0.150	**0.338**	**0.905**
ARGOX	0.201	0.257	**0.360**	**0.160**	**0.170**	0.173	0.283	**0.355**	0.177	0.756	0.133	0.345	0.978
VAR1	1.092	1.202	1.406	0.841	0.793	1.250	1.161	0.905	1.391	2.641	0.339	1.197	4.388
GFT	–	–	–	0.465	0.487	–	–	–	–	–	–	–	–
naive	0.240	0.303	0.421	0.180	0.188	0.207	0.273	0.466	0.246	1.105	**0.069**	0.365	1.100
Correlation													
ARGO-C	**0.978**	**0.968**	**0.964**	0.958	0.950	**0.959**	**0.922**	**0.970**	**0.975**	0.912	0.363	**0.907**	**0.947**
ARGOX	0.976	0.967	0.963	**0.960**	0.952	0.956	0.904	0.969	0.970	**0.916**	0.375	0.893	0.939
VAR1	0.804	0.715	0.734	0.824	0.771	0.584	0.571	0.854	0.498	0.655	-0.005	-0.215	0.636
GFT	–	–	–	0.953	**0.955**	–	–	–	–	–	–	–	–
naive	0.959	0.955	0.953	0.939	0.929	0.929	0.902	0.927	0.929	0.879	**0.696**	0.815	0.909

Table S10-51. Comparison of different methods for state-level %ILI estimation in New York NY. The MSE, MAE, and correlation are reported. The method with the best performance is highlighted in boldface for each metric in each period.
